# Scorpion Envenomation: An Intensive Care Unit Transfer Prediction Score

**DOI:** 10.1590/0037-8682-0052-2026

**Published:** 2026-07-03

**Authors:** Matheus Henrique Leite e Silva, Juliana Sartorelo Almeida, Stefania Villela Moreira Reis, Luiz Eduardo Leverentz Souto, Rafael Lopes de Souza Alves, Heloísa Guimarães Alves Lopes, Marcus Vinícius de Melo Andrade

**Affiliations:** 1 Universidade Federal de Minas Gerais, Belo Horizonte, MG, Brasil.; 2 Fundação Hospitalar do Estado de Minas Gerais, Centro de Informações e Assistência Toxicológica de Minas Gerais, Belo Horizonte, MG, Brasil.

**Keywords:** Scorpion sting, Predictive factors, Prognostic, Intensive care, Envenomation, Nomogram

## Abstract

**Background:**

Scorpion envenomation frequently requires critical care intervention. Early determination of cases with a higher likelihood of clinical deterioration may allow for resource optimization and improvement of clinical endpoints. In this study, patients from a tertiary hospital with similar clinical characteristics but diverse outcomes were analyzed to propose a clinical deterioration prediction score.

**Methods:**

Demographic, clinical, laboratory, and imaging findings of patients with scorpion envenomation were retrieved from a database used in a toxicology center based on in-person attendance data from 2019 to 2024. Multivariate models were defined and evaluated using receiver operating characteristic curves. A nomogram was created to estimate the probability of ICU transfer. Optimal cutoff points, sensitivity, and specificity were computed. Multiple imputation was performed using chained equations. Normality of errors in linear regressions was assessed via QQ plots.

**Results:**

Overall, 146 patients were eligible for enrolment. Sex (*p* = 0.06), weight (*p* = 0.30), time-to-serum (*p* = 0.89), and exposure area (*p* = 0.49) were comparable between patients admitted to the intensive care unit (ICU) and other inpatients, whereas age (*p* = 0.006) and initial severity (*p* < 0.001) displayed significant differences. Logistic regression models displayed area-under-the-curve metrics ranging from 65% to 93%, with high specificity. Hyperamylasemia (*p* = 0.004), vomiting (*p* = 0.006), and cardiac dysfunction on echocardiography or point-of-care ultrasound (*p* < 0.001) were strongly associated with critical care.

**Conclusion:**

Prognostication of factors leading to ICU transfer from initial clinical and laboratory findings is feasible. The regression model achieved high specificity and moderate sensitivity; being a potentially useful confirmatory test in cases with clinical suspicion.

## INTRODUCTION

Scorpions have inhabited the planet since prehistoric times, predating humans in the colonization of territories that are now often shared by both^1^. In Brazil, the Buthidae family is the only group capable of causing severe envenomation, primarily through scorpions of the genus *Tityus*, among which *T. serrulatus* is associated with the highest lethality^2^. 

Myriad risk factors have been proposed in the literature to support the accurate classification of patients after scorpion stings. In cases involving *Tityus serrulatus*, accidents occurring in patients below 10 years of age were previously shown to have the highest severity, with a mean monotonic increase of 13% in mortality for each one-year decrease in age^3^. Another study identified an age threshold of 5 years, below which the probability of clinical deterioration increased by 158% compared with older patients^4^. This vulnerability may stem from the rate between inoculated venom volume and total body surface area in younger children, whilst also being affected by cardiovascular, autonomous nervous system and immunologic immaturity^5^ ^,^ ^6^. 

In countries where scorpion stings represent a public health concern, the incidence ranges from approximately 20 to 400 cases per 100,000 inhabitants^1^ ^,^ ^7^. In Brazil, the progressive increase in cases over the last 10 years has reached a cumulative rise of 349%, with an incidence of 142 cases per 100,000 inhabitants in 2024^8^. Regarding mortality, 1,667 deaths were reported in the country from 2012 to 2024. Although the overall lethality rate was 0.3%, it reached 1.3% among children aged ≤10 years^9^.

Several avoidable factors contribute to deaths and complications associated with scorpion envenomation^10^. Among these, two main barriers are of particular relevance: 1) the lack of familiarity with the symptoms of severe scorpionism, which may hinder early recognition of severity, especially in children, and delay antivenom administration because of delayed hospital referral or the unavailability of the antivenom serum nearby; and 2) the limited access to intensive care unit (ICU) beds, including the long distances often required to access them^10^. 

This study aimed to develop a severity prediction score based on clinical symptoms, laboratory, and sonographic abnormalities using data from in-person care at a reference center to support earlier diagnosis, treatment, and referral of patients with severe scorpion envenomation.

## METHODS

### Study population and setting

This retrospective study used data from DATATOX, an information system of the Brazilian Association of Toxicology Information and Assistance Centers (ABRACIT) used by Toxicology Assistance Centers to register, monitor, store, process, and retrieve data on poisonings and accidents caused by venomous animals in Brazil. Data were collected from the Toxicology Information and Assistance Center of Minas Gerais (CIATOXMG) between 2019 and 2024. For a statistical power of 95%, expecting a minimum Spearman’s correlation coefficient of ρ = 0.3, the required sample size was calculated as 139 patients.

### Inclusion criteria

Patients of any age were eligible if they presented, in person, with moderate or severe scorpion envenomation at the João XXIII hospital (a tertiary care center where CIATOXMG is located), and had been registered in DATATOX. 

### Ethics approval and protocol registration

The study project was approved by the Ethics Committee of the Minas Gerais Hospital Foundation and registered on Plataforma Brazil, under registration number CAAE: 40930820.8.0000.5149. 

### Studied variables

For the predictive analysis, several factors were initially examined through exploratory analysis and subsequently refined using both data-driven and clinical hypothesis-driven frameworks. Clinical variables, laboratory findings, and imaging examinations were evaluated, together with demographic characteristics, length of stay in the ICU, treatments administered, complications, and outcomes. The complete list of variables is provided in Supplementary Table 1S.

Data were reviewed for potential confounders, including comorbidities, superimposed conditions, and other types of envenomation. Clinical presentations potentially attributable to causes unrelated to scorpion envenomation were excluded through chart review. All data extracted from the database were cross-referenced with the patients’ medical records in the hospital electronic record systems.

### Statistical analyses

R version 4.4.2 (R Foundation for Statistical Computing, Vienna, Austria) was employed for statistical analysis^11^. The threshold for statistical significance was pre-defined as *p* < 0.05. The dplyr, mice, ggpubr, Hmisc, corrplot, pROC, scorecard, datadist and rms packages were used.

### Primary outcome

The primary outcome for this study was any clinical deterioration requiring ICU transfer.

### Exploratory analysis

Standard descriptive statistics were run for all variables, including Mann-Whitney-Wilcoxon tests (for non-normally distributed data), t-tests (for normally distributed data), and Fisher’s exact tests to compare characteristics between patients grouped by outcome. 

Correlation matrices via Spearman’s ρ were constructed to assess collinearity between variables, as this step is essential to avoid multicollinearity in regression models. When two highly correlated variables were identified, the variable with greater clinical utility was retained. Clinical utility was defined according to the validity, measurability, and reproducibility of the variable in routine clinical practice. For example, if dyspnea and tachypnea were highly correlated and adding both to the regression model resulted in no increased performance, tachypnea would be chosen, considering its more objective nature.

### Multiple imputation

Regression models require complete data for all independent variables across observations to preserve the effective sample size and statistical power equivalent to that number of observations. Therefore, to minimize data loss, missing values were handled using multiple imputation by chained equations (MICE). This method analyzes the patterns within the dataset to reproduce several versions of likely values for each missing cell. These bootstrapped values are then averaged to generate the final imputed dataset. MICE is known for preserving the underlying data distribution, parameter estimates, uncertainty, and variability, provided that perfect prediction is absent^12^. Analyses were conducted both with and without multiple imputation, and both sets of results are reported.

### Nomogram development process

Based on the exploratory analyses and prior clinical knowledge provided by the senior authors, several regression models were tested while accounting for statistical constraints. Multiple models were required because of the limited degrees of freedom, which were directly related to the sample size. Even with a moderate number of observations (n = 146), it was not feasible to test all variables simultaneously. Selecting high-yield variables allowed models to be more practical and intuitive while preserving statistical robustness.

Exploratory logistic regression models were constructed, each with 5-10 variables, including the dependent variable, ICU transfer. Univariate and multivariate linear and logistic regression models were built in the preliminary analyses and nomogram development process. For linear regression models, the assumption of normally distributed errors was indirectly assessed through residual analysis conducted with QQ plots, Shapiro-Wilk tests, and Kolmogorov-Smirnov tests, with priority given to visual assessment. The initial regression lines and distribution analyses are presented in the Supplementary Appendix. Receiver operating characteristic (ROC) curves were generated for each model, area under the curve (AUC) metrics were compared, and optimal cut-off points were calculated. For logistic regressions, Wald tests were used to evaluate the strength and statistical significance of associations.

For the multivariate analysis, symptoms were grouped according to the affected system as neurologic, cardiac, respiratory, or gastrointestinal.

The model with the highest AUC was converted into a nomogram, a clinical decision-support tool. The nomogram estimates the probability of ICU transfer at admission based on the clinical, laboratory, and imaging findings identified in this study.

## RESULTS

### Baseline characteristics

**FIGURE 1:**
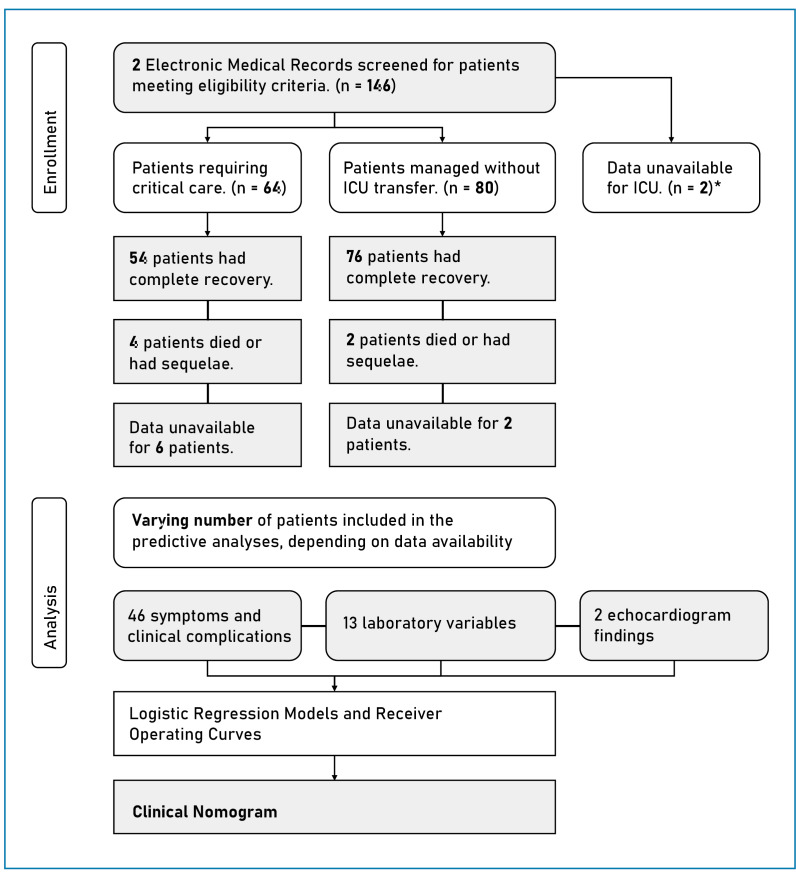
Study Flow Diagram. **ICU**: Intensive care unit.

As shown in Table 1, 146 patients were eligible for enrollment from 2019 to 2024. Sex, weight, time-to-serum and exposition site were comparable between patients admitted to the ICU and other inpatients, whereas age and initial severity displayed significant differences. ICU patients were younger (mean [SD]: 4.763 [3.867]; 11.407 [15.521]; *p* = 0.006) and had a more severe initial classification (ICU: mild 0%; moderate 14.06%; severe 85.93%; Not-ICU: mild 6.25%; moderate 37.5%; severe 56.25%; Fisher’s exact test, *p* < 0.001). Regarding survival outcomes, no baseline variables differed significantly between survivors and nonsurvivors. The study flow diagram is depicted in Figure 1.

**TABLE 1:** Baseline Characteristics.

Variable	All patients	ICU Transfer^+^		***p*-values**	Complete recovery*		***p*-values**
	(n = 146)	Yes (n = 64)	No (n = 80)		Yes (n = 135)	No (n = 6)	
Age (median [min-max])	4.5 [0.25-74]	4 [0.25-17]	6 [0.67-74]	0.006	5 [0.67-74]	3 [0.25-12]	0.1998
**Sex (n)**							
**Male**	71	26	46	0.064	69	2	0.6807
**Female**	70	38	34		66	4	
**Weight** **(median [min-max])** (n = 79)**	16 [5.7-87]	15.25 [5.7-87]	17 [8.8-60]	0,30	17 [5.7-87]	15 [6-15.5]	0.2252
**Initial Severity (n)**							
**Low**	5	0	5	< 0.001	5	0	0.3440
**Intermediate**	40	9	30		39	0	
**High**	101	55	45		91	6	
**Exposition Area (n)**							
**Urban**	118	51	65	0.499	110	3	0.5277
**Rural**	23	12	11		22	1	
**Time to Anti-scorpionic Serum (minutes) (n = 141)** ^++^ **(median [min-max])**	105 [25-840]	110 [30-600]	90 [25-840]	0.891	102.5 [25-840]	120 [60-360]	0.2858

*Five patients had no information regarding sequelae-free survival. ** Weight was only available for 79 patients. ^+^ Two patients had no information regarding ICU transfer. Two patients had no information regarding time to serum. Mann-Whitney-Wilcoxon tests (if nonnormal distribution) and Student’s t tests (if normal distribution) were used for continuous variables and Fisher’s exact tests were conducted for discrete data. Median and ranges (minimum and maximum values in the sample) are given when data was non-normally distributed. Mean and standard deviation data are available in the Supplementary Appendix.

Although the specific *Tityus* species involved in each case was not explicitly identified, *Tityus serrulatus* is known to account for approximately 97% of scorpion stings in the region where this study was conducted. Moreover, envenomations by other *Tityus* species, such as *T. stigmurus* and *T. bahiensis*, which are also prevalent in the region, present with a similar overall symptomatic profile as those caused by *T. serrulatus*. However, these accidents typically result in a lower incidence of severe clinical outcomes when compared to *T. serrulatus* stings.

### Laboratory findings

When patients were stratified by ICU referral, those transferred to the ICU had significantly higher platelet counts (*p* = 0.04) and leukocyte counts (*p* = 0.02). In addition, pH (*p* = 0.07) and amylase levels (*p* = 0.09) displayed a trend towards significance (Table 2).

**TABLE 2:** Laboratory Findings.

Variable	All patients	ICU Transfer^+^		***p*-values**	Complete recovery*		***p*-values**
	(n = 146)	Yes (n = 64)	No (n = 80)		Yes (n = 135)	No (n = 6)	
**RBCs (n = 101)**	4.484	4.55	4.409	0.284	4.505	4.356	0.6683
(mean [SD])	[0.457]	[0.461]	[0.447]		[0.442]	[0.711]	
**WBCs (n = 103)**	16765.728	18043	14876	0.025	16999.891	15248.333	0.5988
(mean [SD])	[6457.355]	[6391]	[6170]		[6501.670]	[5952.506]	
**PLT (n = 102)**	357313	378847	329452	0.043	364945.055	265833.333	0.1753
(mean [SD])	[108367]	[103774]	[109484]		[104359.567]	[162516.973]	
**K** ^+^ **(n = 111)**	3.6	3.6	3.75	0.554	3.6	3.8	0.3210
(median [min-max])	[2.3-5.9]	[2.6-5.9]	[2.3-5.9]		[2.3-5.5]	[2.9-5.9]	
**Na** ^+^ **(n = 110)**	141	140	141	0.629	141	140	0.1919
**(median [min-max])**	[127-150]	[132-147]	[127-150]		[127-150]	[139-141]	
**BIC** **(n = 84)** **(median [min-max])**	18.7 [6.9-25.2]	18 [6.9-25.2]	18.9 [7-24]	0.155	18.6 [7-25.2]	18.55 [6.9-22.8]	0.8710
**Total CPK (n = 93)**	277	278	275.5	0.646	249	2134.5	0.004
**(median [min-max])**	[60-21252]	[60-3109]	[79-21252]		[60-21252]	[324-14109]	
**Troponin (n = 102)**	4.37	6.63	4.16	0.852	3.765	2751	0.0012
**(median [min-max])**	[0.012-30000]	[0.012-13740]	[0.012-30000]		[0.012-30000]	[27.4-13740]	
**Amylase (n = 87)**	132	155	118	0.090	138.5	114	0.3210
**(median [min-max])**	[34-1007]	[46-1007]	[34-683]		[34-1007]	[62-175]	
**Lactate (n = 84)**	3.45	3.8	3.2	0.209	3.6	1.75	0.1367
**(median [min-max])**	[0.6-23]	[0.6-8.3]	[0.8-12.8]		[0.6-23]	[1.1-5.5]	
**pH (n = 84)**	7.395	7.41	7.376	0.074	7.394	7.395	0.7520
**(mean [SD])**	[0.082]	[0.072]	[0.091]		[0.081]	[0.111]	
**Serum Glucose (n = 97)**	137	140.5	134	0.913	149	118	0.6419
**(median [min-max])**	[59-409]	[60-409]	[59-388]		[59-409]	[60-287]	
**Capillary Glucose (n = 64)**	242	246	237.5	0.354	243	243	1
**(median [min-max])**	[79-500]	[87-440]	[79-500]		[79-500]	[96-390]	

* Five patients had no information regarding sequelae-free survival. Mann-Whitney-Wilcoxon tests (if nonnormal distribution) and Student’s t-tests (if normal distribution) were used for continuous variables. The median and ranges (minimum and maximum values in the sample) are given when data was non-normally distributed. Mean and standard deviation data are available in the Supplementary Appendix.

### Individual symptom analysis

In the univariate analysis evaluating individual symptoms, only manifestations that clearly reflected advanced severity and tended to appear late in the pathological process, such as acute pulmonary edema, were significantly associated with survival outcomes. The univariate symptom analysis is presented in Supplementary Table 2S **.**

### Exploratory analyses

The correlation matrices showed an overall low correlation between variables, although some pairs displayed significant values. Only baseline clinical characteristics and laboratory findings were included in the correlation analysis because of the increasing complexity of handling continuous and discrete variables within the same correlation matrix. Significant correlation was defined as Spearman’s ρ > 0.7 or < -0.7.

Age and weight (ρ = 0.81), and total creatine phosphokinase and troponin (ρ = 0.74) were highly correlated. Based on the prespecified thresholds, no variables showed significant negative correlations. However, serum glucose and potassium (ρ = −0.49), age and platelet count (ρ = −0.47), and white blood cell count and potassium (ρ = −0.46) showed trends towards significance. The full correlation table is available in the Supplementary Appendix.

### Univariate regression models

Besides correlation analyses, variables were also tested on their direct relationship to the outcomes. Several linear and logistic regression models were developed. For the prediction of survival outcomes, most logistic regression curves were nonsignificant. Notably, lower platelet counts were significantly associated with death or sequelae (*p* = 0.040). Additionally, troponin (*p* = 0.075) and CPK (*p* = 0.061) levels displayed trends towards significance. A subjective severity-change variable was used as a positive control in this analysis and was significantly associated with survival outcomes (*p* < 0.001). However, the rarity of the death or sequelae outcome impacted statistical power and may limit the reliability of these associations.

Using univariate ROC curves, shown in Supplementary Figure 1S, the optimal thresholds were identified as 166,000 platelets/µL, troponin levels of 861.75 ng/L, and total CPK values of 1,362 UI/L. For the prediction of death or sequelae, lower platelet counts showed a specificity of 0.978, although low sensitivity of 0.500. CPK levels showed specificity of 0.963 and sensitivity of 0.667, whereas troponin levels displayed more balanced predictive accuracy, with specificity of 0.856 and sensitivity of 0.833.

Symptoms and laboratory findings did not reliably predict ICU transfer in the univariate analysis. Only vasoactive amine use (*p* = 0.003) and diuretic use (*p* = 0.011) were significantly associated with the primary outcome. pH displayed a trend towards significance after adjustment (unadjusted *p* = 0.113; adjusted *p* = 0.0502), and bicarbonate also showed a near-significant association (*p* = 0.06). Intriguingly, cardiac dysfunction on echocardiography or point-of-care ultrasound (POCUS) did not predict ICU transfer. The unadjusted and adjusted univariate regression analyses are reported in the Supplementary Appendix.

### Multivariate clinical prediction models

Four multivariate models were initially developed based on prior clinical knowledge and then analyzed to refine a fifth prediction model.

Afterwards, because missing data could lead to substantial information loss and potentially under or overestimate model accuracy, missing observations were resampled via multiple imputation by chained equations. Overall, 12.818% of total observations were imputed. For the fifth model, for example, only 46 observations, including 40 events and six controls, were simultaneously available for all covariates. This could compromise model validation and potentially overestimate performance indicators, such as AUC, and was mitigated via multiple imputation.

The five-fold imputed datasets were then used to validate the fifth model. Before refitting, the mean AUC for model 5 across the five imputed datasets was 0.648 ± 0.032.

Among the included variables, cardiac dysfunction on echocardiography or POCUS and glycemia displayed lower coefficients and higher *p*-values, suggesting limited contribution to the model’s predictive performance. Considering, however, that cardiac dysfunction is clinically relevant in severe scorpion envenomation, this finding suggested a potential inconsistency in the model’s logic. 

Upon refitting the model, using the same variables, on the imputed dataset, the sixth model showed moderate accuracy, with AUC values ranging from 0.77 to 0.83, alongside moderate Akaike Information Criterion (AIC) values. Further analysis led to the development of a seventh model, which integrated findings from previous models and the univariate analyses while also ungrouping symptom covariates to improve interpretability and simplify the model structure. This model was fit and validated using the imputed dataset and showed improved predictive performance, with an AUC of 0.93 and an AIC of 137.

A simplified version of the seventh model was generated by decreasing the number of covariates to improve clinical practicality, considering the nomogram development process. The covariates for the final model and some statistical parameters were (simplified seventh model covariates): cardiac dysfunction on echocardiography or POCUS, tachypnea, cold extremities, drowsiness, pulmonary crepitus, vomiting, abdominal pain, sweating, tachycardia, bradycardia, hypotension, low oxygen saturation, sodium, amylase, and lactate. This model had an AIC of 130.2 and an AUC of 0.92. The optimal cut-off point was 0.567 (calculated probability), corresponding to a specificity of 0.95 and a sensitivity of 0.78.

**FIGURE 2:**
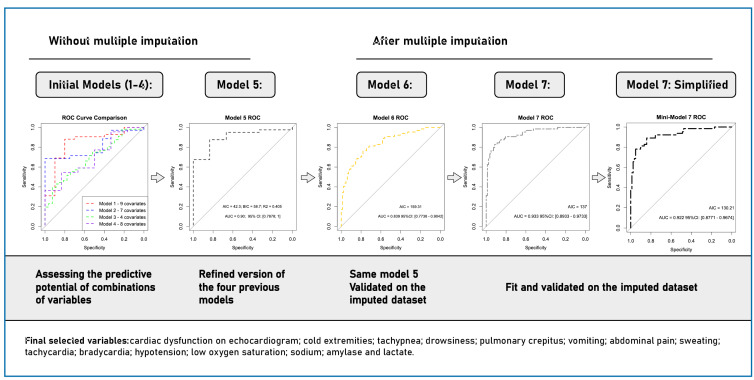
Exploratory analyses - Logistic regression models and ROC curves. **AIC:** Akaike Information Criterion; **BIC:** Bayesian Information Criterion; **AUC:** Area Under Curve; **CI:** Confidence Interval; **ROC:** Receiver Operating Curve.

The ROC curves are shown in Figure 2. All model variables are described in the Supplementary Appendix.

### Nomogram

**FIGURE 3:**
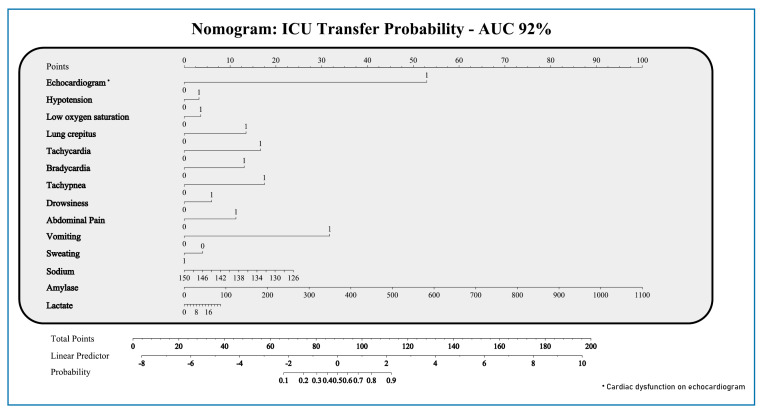
Preliminary ICU transfer prediction nomogram. *Cardiac dysfunction on echocardiography.

Using the rms package in R, the calculated regression coefficients were proportionally converted into a visual scoring system. Through this process, the simplified model 7 was converted into a nomogram - a graphical clinical score, shown in Figure 3.

### Application

To illustrate the potential clinical application of the nomogram, we present two fictitious examples. 1) J.P.S., a seven-year-old female patient, was referred to the hospital with hypotension, tachycardia, profuse vomiting, tachypnea, and somnolence. Initial laboratory workup showed serum amylase of 350 U/L, serum sodium of 134 mEq/L, and lactate of 10 mmol/L. Respiratory and cardiovascular auscultation revealed no marked abnormalities. POCUS showed no obvious systolic or diastolic cardiac dysfunction. The patient received antivenom a few minutes after admission. Although she initially had few clinical signs of severity (severe cardiac, neurological, or respiratory instability), her condition deteriorated with signs of shock and respiratory distress, requiring inotropic agents and non-invasive ventilation. After two hours, with poor response to therapy, the patient was transferred to the ICU, where she received critical care for 4 days, and was discharged without sequelae in 7 days, counted from admission. At admission, before clear clinical signs of instability were present, the nomogram estimated a 95% probability that critical care would be required, corresponding to 222 points.

2) A.S.O., a six-year-old male patient, was referred with a similar clinical picture but without tachypnea or lethargy and with pulmonary crepitus. Application of the clinical score yielded a value of 152, representing an estimated probability of 80% that critical care would be necessary. The patient was not promptly transferred to the ICU, considering the simultaneous presence of another patient with a more severe clinical picture. Three hours after adjuvant therapy and antivenom administration, he regained hemodynamic stability and continued to be closely monitored for any additional clinical change for the following days. He was discharged two days after admission.

In this example, the nomogram supported clinical decision-making by allowing for a subsequent patient stratification in a scenario where both appeared severely ill, assuming that the required clinical and laboratory data were available for both patients at the time of assessment.

A multimedia example of nomogram application, developed with artificial intelligence integration and corresponding to patient J.P.S., is available in the Supplementary Appendix.

## DISCUSSION

### Summary of main findings

It is possible to predict, with relatively high accuracy, which cases of scorpion envenomation will require ICU transfer based on initial presentation symptoms, imaging, and laboratory results. This approach may allow for prompt identification of patients likely to deteriorate at an early clinical stage, ultimately decreasing time to ICU transfer decisions and increasing vigilance for potential complications.

In this study, 6 years of compiled data from patients diagnosed with scorpion envenomation were analyzed and translated into a clinical prediction tool. Although still lacking validation in larger databases and further adaptation according to local resource availability, this framework may help establish a hierarchy of clinical developments and presentations which could be useful in both clinical and research scenarios.

### In context with the literature

As of writing, this is the first nomogram designed to predict ICU transfer in patients with scorpion envenomation. Previous works have primarily focused on prognostic factors associated with mortality and complications among patients already admitted to the ICU, such as the Pediatric Risk of Mortality score (PRISM III)^13^, or aimed at the development of clinical severity grading systems, such as the Abroug scale and hospitalization scores. Although these tools are not directly related to critical care, they may serve as surrogate methods for assessing disease severity^14^ ^-^ ^16^.

The role of cardiac evaluation by ultrasound as a marker of severity is well established in the literature and was supported by the findings of this study as well^17^ ^,^ ^18^. 

Regarding the symptoms included in the score, tachypnea and pulmonary edema may reflect a direct consequence of increased pulmonary vascular permeability, hypoxia, and metabolic acidosis. In scorpion envenomation, respiratory distress may originate from autonomic dysfunction, pulmonary edema, or direct venom-mediated effects on inflammatory cytokines^6^ ^,^ ^19^. 

Cold extremities may indicate peripheral vasoconstriction resulting from sympathetic stimulation or shock. Scorpion venom can induce catecholamine release, leading to vasoconstriction and, particularly in children, the assessment of extremity perfusion is an important clinical sign associated with shock and overall severity^20^. 

Symptoms such as vomiting, abdominal pain, sweating, and increased gastric motility are common after envenomation, primarily because of cholinergic stimulation and the systemic inflammatory response induced by the venom. Studies in pediatric populations demonstrate that the presence and intensity of gastrointestinal symptoms are associated with greater clinical severity and increased mortality risk. These symptoms have been identified as independent predictors of poorer prognosis in multivariate analyses^21^ ^-^ ^22^. 

Hyperamylasemia, the laboratory finding most strongly associated with ICU transfer in the final regression model, has also been pointed out as a relevant biomarker in previous works^23^ ^,^ ^24^. The significance of hyperamylasemia in the context of severe scorpion envenomation is primarily as a marker of neurohormonal stimulation and acute pancreatic inflammation. It does not necessarily indicate structural acute pancreatitis but may instead represent a transient response to the systemic toxicity of the venom^22^. 

### Generalizability

Although this study was conducted in a region where *Tityus serrulatus* is the predominant cause of severe scorpion envenomation, the findings may be generalizable to other medically important scorpion species in the Buthidae family. This generalizability is supported by the shared pathophysiology of severe scorpion envenomation. Systemic effects are largely mediated by venom components that induce massive catecholamine release, often referred to as 'catecholaminergic storms', and trigger a systemic inflammatory response, leading to similar clinical presentations across different scorpion species^18^ ^,^ ^20^ ^-^ ^22^ ^,^ ^25^. This approach aligns with international consensuses, such as the classification developed by Khattabi et al. (2011), which deliberately adopted a species-agnostic framework^26^. Their classification emphasized that severe clinical outcomes are primarily defined by the patient’s physiological response rather than by the specific venom-producing species. Accordingly, our prediction score, which is based on observable clinical signs, laboratory parameters, and echocardiographic findings, focuses on universal markers of systemic toxicity. This may allow early identification and risk stratification of severe cases, facilitating timely and appropriate management that transcends geographical specificities.

### Implications and limitations

None of the reported models can perfectly determine whether a patient will require ICU transfer based solely on early variables. Nevertheless, the proposed nomogram may allow for accurate refinement of clinical judgement and support decision-making, particularly in resource-constrained settings or in patients with atypical presentations.

Limitations include the lack of complete data availability for all variables, which led to the use of multiple imputation. Although this resampling method is reliable and statistically robust, it should be regarded as a statistical aid, allowing for a more complete analysis rather than a study design improvement tool or a substitute for complete prospective data collection.

The relative rarity of this condition should also be considered. Throughout more than half a decade of functioning, a tertiary hospital with a high patient inflow accumulated 146 eligible patients. This limited sample size may have contributed to higher unexplained heterogeneity and lower goodness-of-fit metrics. A strength of the study, conversely, was the input of toxicologists with decades of experience in the field, thereby contributing to generating, confirming, and refuting hypotheses arising from statistical analyses.

Although validated, the nomogram should not be interpreted as a standalone recommendation for ICU transfer. Clinical experience and judgement should always be multifactorial, and this tool may be only one of these factors, integrating a broader decision-making process. It is reasonable to apply this and other scores to estimate prognosis, being certain that no method is fail-safe and that decisions should ideally be individualized.

### Conclusion

Severe scorpion envenomation frequently requires critical care intervention and ICU admission. Early determination of which cases have a higher likelihood of clinical deterioration may allow for resource optimization and improve clinical outcomes. The logistic regression-based nomogram developed in this study showed high specificity and moderate sensitivity, suggesting potential utility as a confirmatory test to support clinical decision-making.

Although the nomogram was primarily developed using data from patients stung by *Tityus serrulatus*, the observed clinical and laboratory alterations may be extrapolated to envenomations caused by other species within the Buthidae family, given their shared pathophysiology and highly consistent clinical manifestations.

## Data Availability

Research data is available upon reasonable request.

## SUPPLEMENTARY MATERIAL

Supplementary Table 1S

Supplementary Table 2S

Supplementary Figure 1S
