# Supplementary material for: Scorpion Envenomation: An Intensive Care Unit Transfer Prediction Score
Source: Rev Soc Bras Med Trop. 2026 Jul 3;59:e0052-2026. doi: 10.1590/0037-8682-0052-2026 (PMC13331192; doi:10.1590/0037-8682-0052-2026)
Supplement: Supplementary Table 1S [file 1678-9849-rsbmt-59-e0052-2026-md1.pdf]

2. Supplementary Tables

2.1 Supplementary Table 1S: Variable List

**Variable**
**Symptoms** (one variable each): pain, tremors, sweating, paleness, nausea, vomiting, sialorrhea, abdominal pain, irritability, drowsiness, cold extremities, tachypnea, bradycardia, tachycardia, dyspnea, rales, low oxygen saturation, hypotension, hypertension, circulatory shock, altered mental status, convulsion, fever, arrhythmias, cardiopulmonary arrest, acute lung edema, infection.
**Laboratory findings** (one variable each): capillary blood glucose, venous blood glucose, leukocytes, erythrocytes, platelets, sodium, potassium, bicarbonate, amylase, pH, lactate, troponin, total CK,
**Imaging:** Cardiac dysfunction on echocardiogram.
**Primary outcome:** Intensive care unit transfer
**Secondary outcomes** (one variable each): intubation, death, sequelae.

A total of 46 variables were included in this analysis, after duplicate variables were discarded.
