# Supplementary material for: Scorpion Envenomation: An Intensive Care Unit Transfer Prediction Score
Source: Rev Soc Bras Med Trop. 2026 Jul 3;59:e0052-2026. doi: 10.1590/0037-8682-0052-2026 (PMC13331192; doi:10.1590/0037-8682-0052-2026)
Supplement: Supplementary Table 2S [file 1678-9849-rsbmt-59-e0052-2026-md2.pdf]

2.2 Supplementary Table 2S: Signs and Symptoms at Initial Presentation – Survival Analysis

Variables (%)	All patients (n = 137)*	Survivors (n = 129)	Nonsurvivors o[illegible] with Severe Disability (n = 6	p-value**
**Vomiting**	90	87	2	0.1799
**Nausea**	19	18	1	1
**Abdominal Pain**	15	15	0	1
**Cold Extremities**	5	5	0	1
**Respiratory Insufficiency**	5	5	0	1
**Tachypnea**	32	29	2	0.6205
**Irritability**	2	2	0	1
**Lethargy/ Drowsiness**	22	22	0	0.5889
**Salivation**	19	19	0	0.5943
**Wheezing**	1	1	0	1
**Diffuse Sweating**	52	49	2	1
**Sphincter Relaxation**	1	1	0	1
**Myosis**	1	1	0	1
**Muscle Spasms**	1	1	0	1
**Oliguria**	1	1	0	1
**Altered Level o[illegible] Consciousness**	6	6	0	1
**Hypotension**	6	5	0	1
**Hypertension**	7	7	0	1
**Acute Pulmonar Edema**	15	12	3	**0.0183**
**Bradycardia**	25	24	1	1
**Tachycardia**	27	26	1	1
**Palpitations**	1	1	0	1

**Hypothermia**	3	3	0	1
**Hyperthermia**	1	1	0	1
**Cardiorespirator Arrest**	1	1	0	1
**Rash**	1	1	0	1
**Hypotonia**	1	1	0	1
**Dizziness**	1	1	0	1
**Paresthesia**	1	1	0	1

*Clinical presentation data not available for 9 patients, with 6 in the Survivors group.
**Hypothesis tests comparing the groups were conducted by Fisher's exact tests.
