# Supplementary figures and images for: Scorpion Envenomation: An Intensive Care Unit Transfer Prediction Score
Source: Rev Soc Bras Med Trop. 2026 Jul 3;59:e0052-2026. doi: 10.1590/0037-8682-0052-2026 (PMC13331192; doi:10.1590/0037-8682-0052-2026)

## 1. Supplementary Figures

### 1.1 Supplementary Figure 1S: Univariate ROC Curves

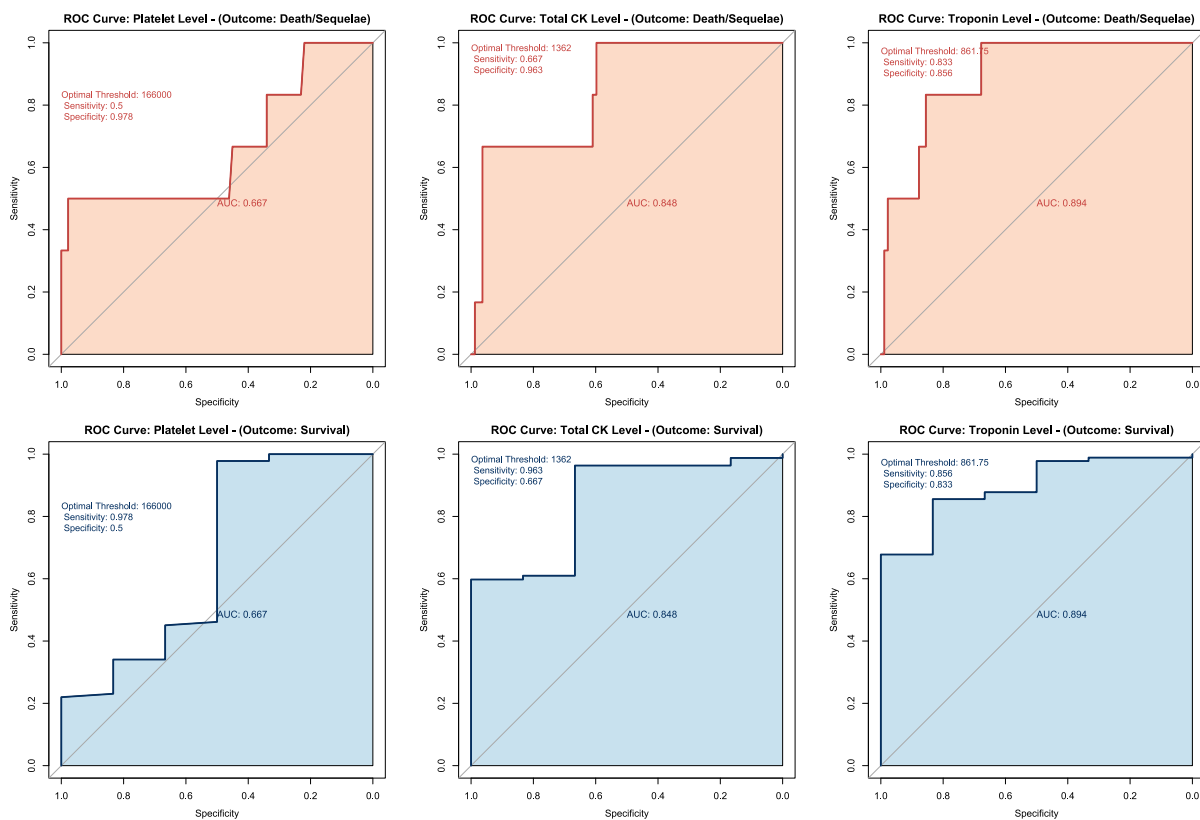

Supplement: Supplementary Figure 1S [file 1678-9849-rsbmt-59-e0052-2026-md3.pdf]
