# Supplementary material for: Scorpion Envenomation: An Intensive Care Unit Transfer Prediction Score
Source: Rev Soc Bras Med Trop. 2026 Jul 3;59:e0052-2026. doi: 10.1590/0037-8682-0052-2026 (PMC13331192; doi:10.1590/0037-8682-0052-2026)
Supplement: Supplementary Appendix [file 1678-9849-rsbmt-59-e0052-2026-md4.pdf]

SUPPLEMENTARY APPENDIX

Scorpion Envenomation: An Intensive Care Unit Transfer Prediction Score

Running title: Scorpion Envenomation: A Prediction Score

Index

1. Supplementary Figures

1.1 Univariate ROC curves

1.2 Correlation Matrix

1.3 Univariate and Multivariate Regressions

1.4 Residual Analyses – Linear Regressions

1.5 Death of Sequelae Univariate and Multivariate Analyses

2. Supplementary Tables

2.1 Suppl. Table 1S. Variable List

2.2 Suppl. Table 2S. Signs and Symptoms at Initial Presentation

2.3 Suppl. Table 3S: Alternative Table 1

2.4 Suppl. Table 4S: Alternative Table 2

2.5 Suppl. Table 5S: Statistical Power Table

3. Model variables

4. Nomogram experimental multimedia example

1. Supplementary Figures

1.1 Supplementary Figure 1S: Univariate ROC Curves

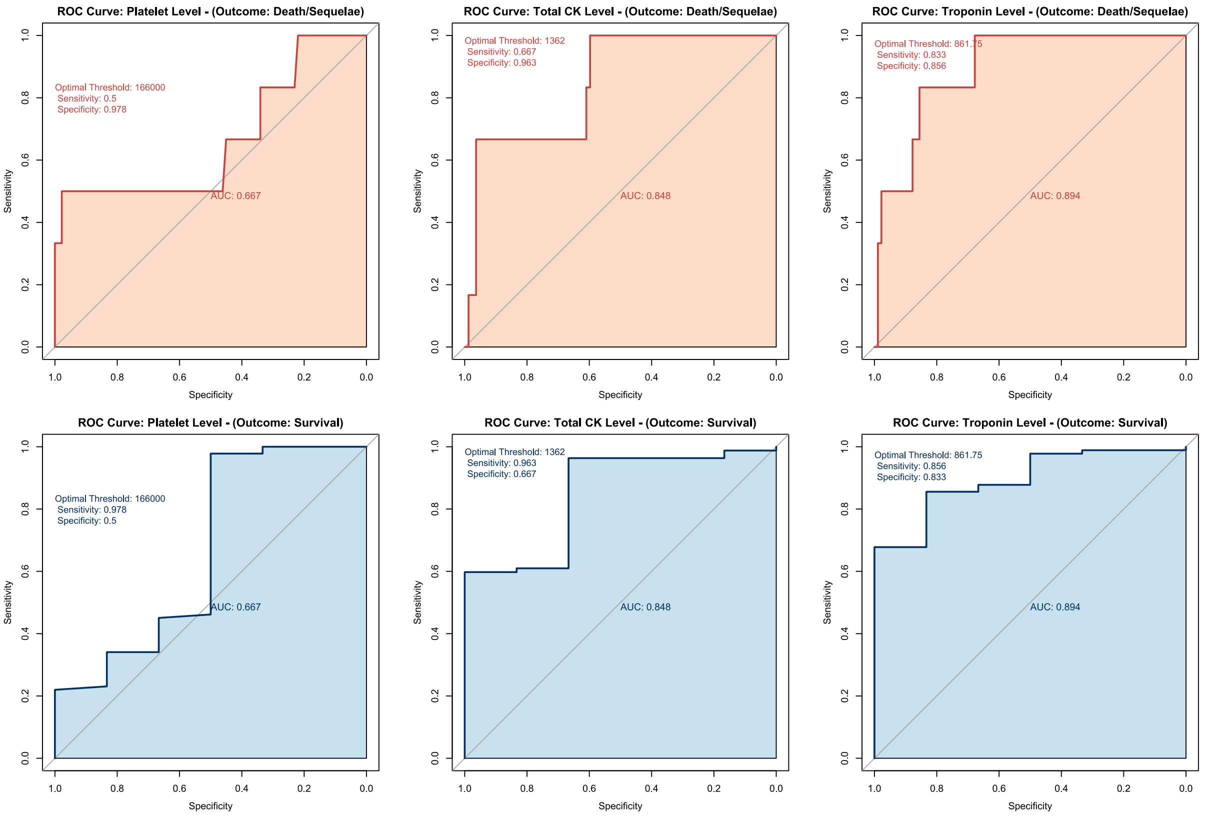

1.2 Supplementary Figure 2S: Correlation Matrix

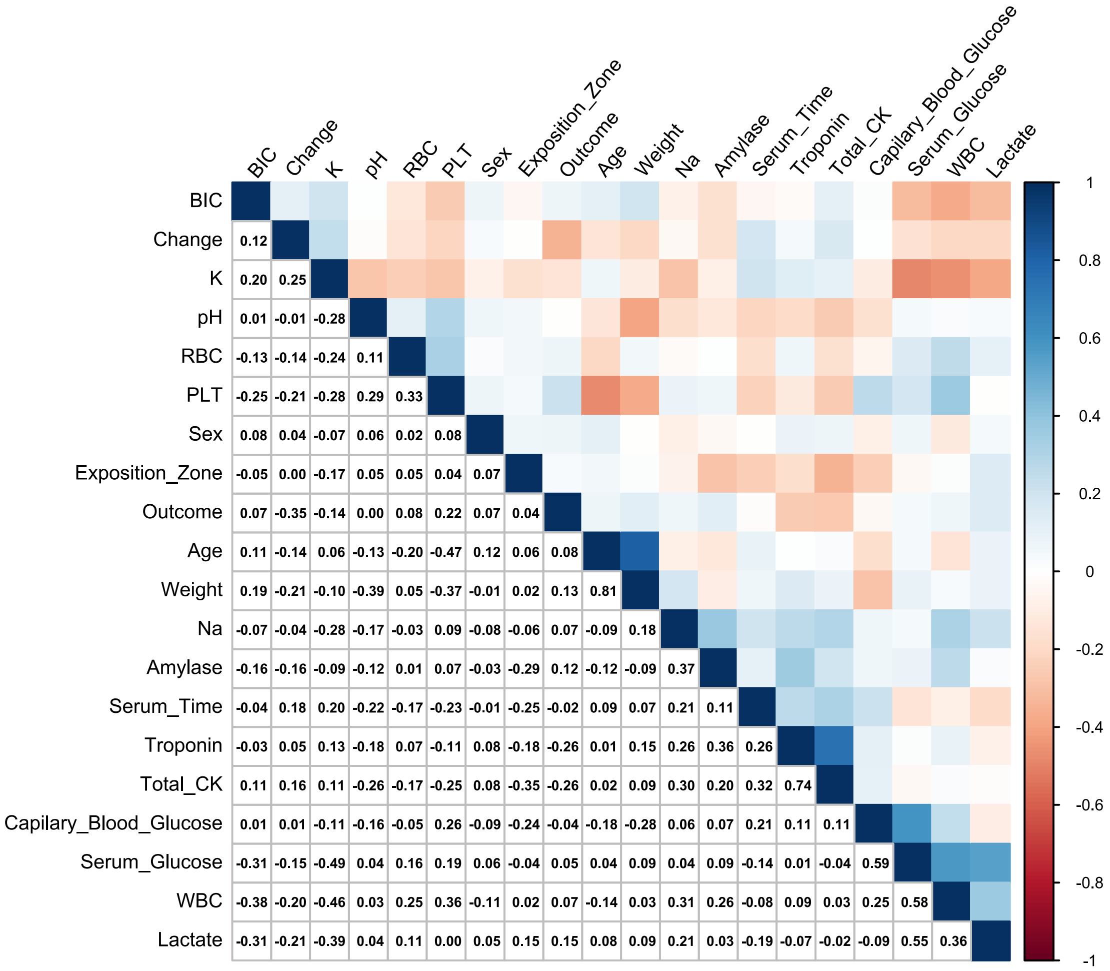

Legend: Change refers to the change in severity as assessed by the primary physician (0 if severity maintained; 1 if severity increased and -1 if severity decreased); Outcome refers to complete recovery from the episode (1) or not (0). BIC: Serum Bicarbonate; PLT: Platelet count; Serum_Time: Time from accident to serum administration; RBC: Red Blood Cell count; WBC: White Blood Cell count. The upper triangle demonstrates Pearson's r correlation values as gradients whereas the lower triangle displays the absolute values.

1.3 Supplementary Figure 3S: Univariate and Multivariate Regressions – ICU Transfer

Respiratory Symptoms

Univariate Model

OR : 2.49
95% CI : [0.77, 7.99]
p-value : 0.1265

P(Intensive Care|0): 0.625
P(Intensive Care|1): 0.806

AUC : 0.611
n : 60

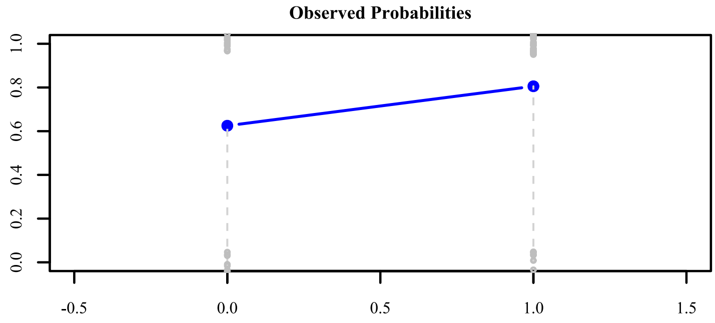

Multivariate Model

Adj OR : 3.15
95% CI : [0.79, 12.59]
p-value : 0.1040
Covariates: 6
AUC : 0.807
AIC : 68.9
n : 60

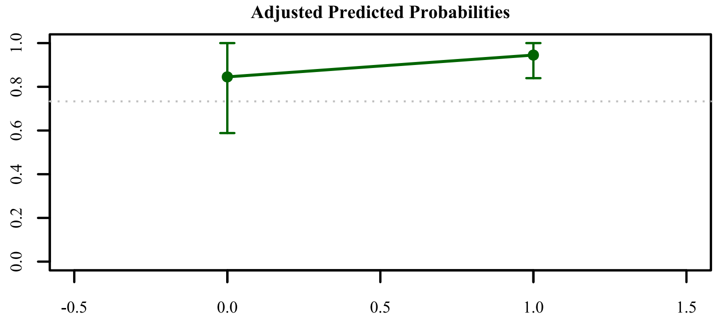

Cardiac Symptoms

Univariate Model

OR : 0.88
95% CI : [0.26, 3.00]
p-value : 0.8365

P(Intensive Care|0): 0.750
P(Intensive Care|1): 0.725

AUC : 0.514
n : 60

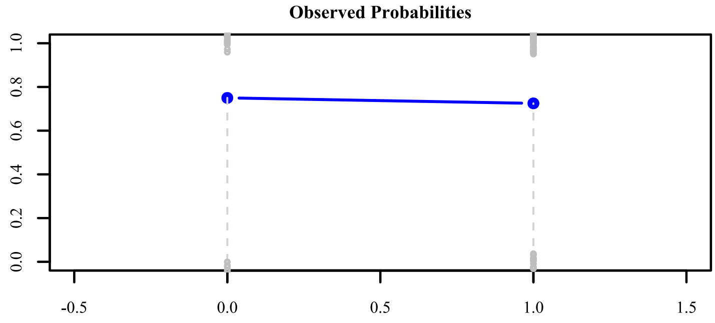

Multivariate Model

Adj OR : 0.65
95% CI : [0.15, 2.82]
p-value : 0.5618
Covariates: 6
AUC : 0.786
AIC : 71.3
n : 60

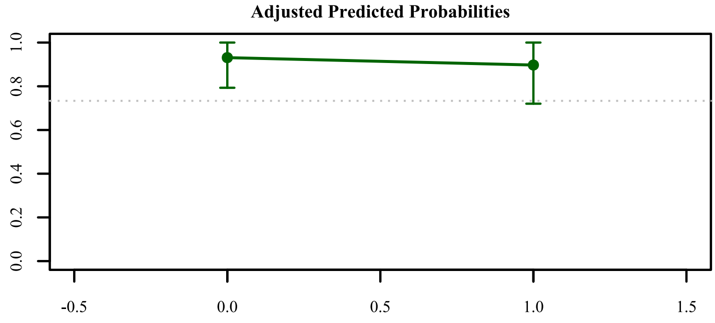

Neurologic Symptoms

Univariate Model

OR : 0.72
95% CI : [0.21, 2.44]
p-value : 0.6003

P(Intensive Care|0): 0.773
P(Intensive Care|1): 0.711

AUC : 0.537
n : 60

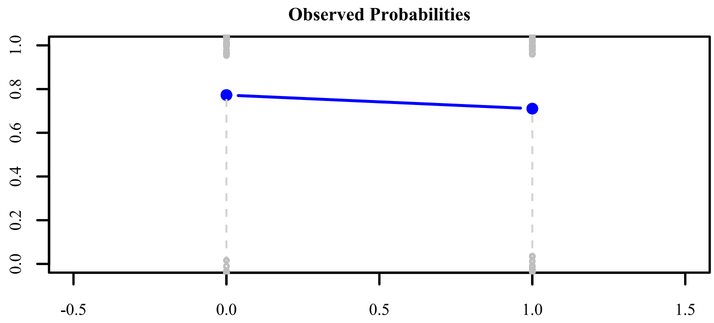

Multivariate Model

Adj OR : 0.91
95% CI : [0.21, 3.95]
p-value : 0.8976
Covariates: 6
AUC : 0.790
AIC : 71.6
n : 60

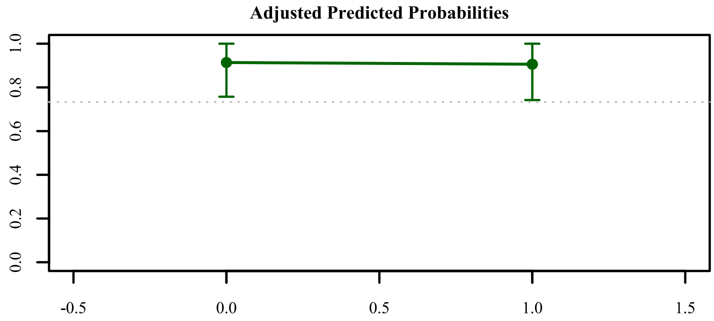

Gastrointestinal Symptoms

Univariate Model

OR : 0.90
95% CI : [0.16, 5.02]
p-value : 0.9089

P(Intensive Care|0): 0.750
P(Intensive Care|1): 0.731

AUC : 0.506
n : 60

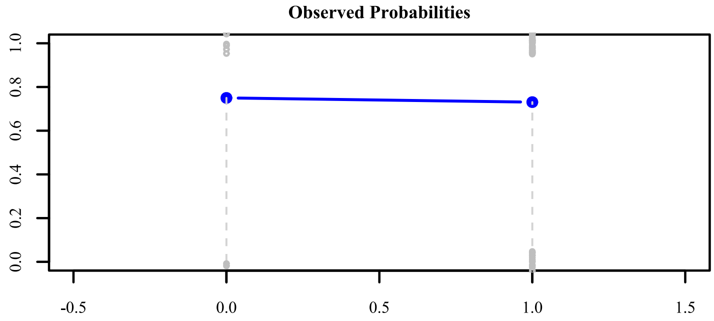

Multivariate Model

Adj OR : 1.37
95% CI : [0.14, 13.06]
p-value : 0.7862
Covariates: 6
AUC : 0.781
AIC : 71.6
n : 60

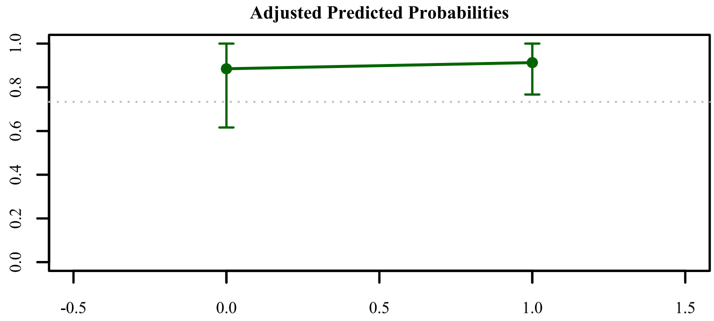

Acute Lung Edema

Univariate Model

OR : 5962423.16
95% CI : [0.00, Inf]
p-value : 0.9927

P(Intensive Care|0): 0.724
P(Intensive Care|1): 1.000

AUC : 0.523
n : 60

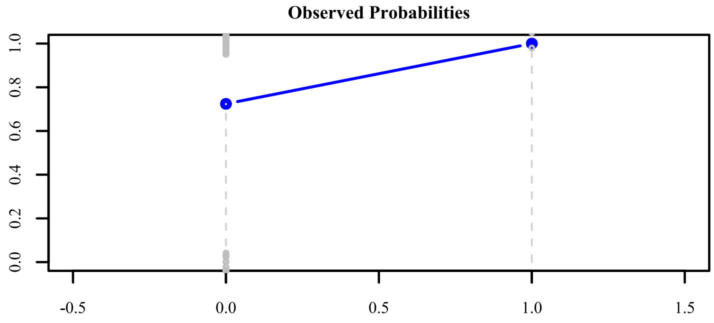

Multivariate Model

Adj OR : 9267444.71
95% CI : [0.00, Inf]
p-value : 0.9922
Covariates: 6
AUC : 0.804
AIC : 70.0
n : 60

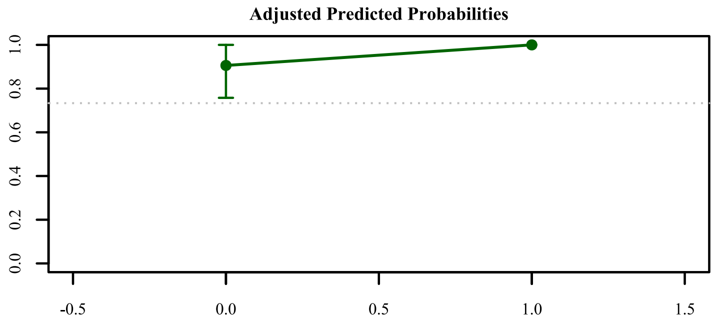

Endotraqueal Intubation

Univariate Model

OR : 17913605.20
95% CI : [0.00, Inf]
p-value : 0.9917

P(Intensive Care|0): 0.704
P(Intensive Care|1): 1.000

AUC : 0.568
n : 60

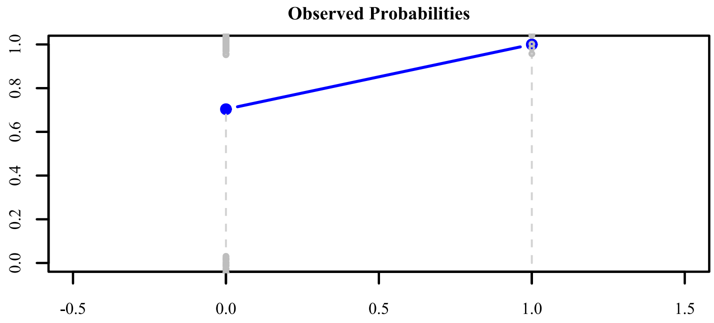

Multivariate Model

Adj OR : 21379674.23
95% CI : [0.00, Inf]
p-value : 0.9922
Covariates: 6
AUC : 0.798
AIC : 68.5
n : 60

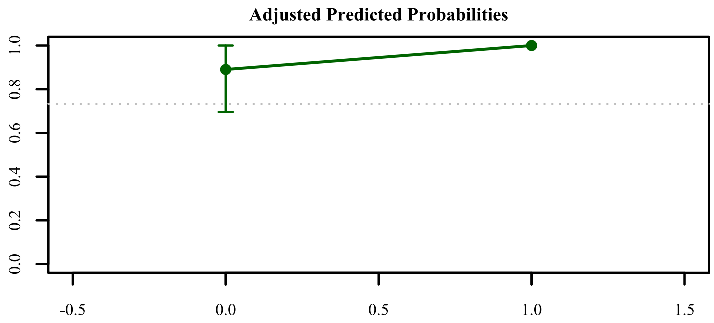

Vasoactive Amine Tx

Univariate Model

OR : 23.82
95% CI : [2.88, 197.14]
p-value : 0.0033

P(Intensive Care|0): 0.531
P(Intensive Care|1): 0.964

AUC : 0.776
n : 60

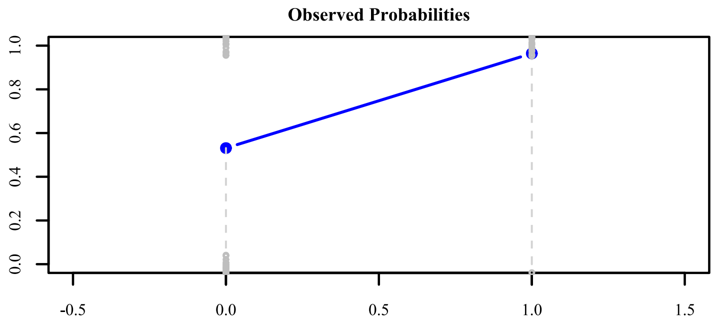

Multivariate Model

Adj OR : 18.98
95% CI : [1.82, 197.58]
p-value : 0.0138
Covariates: 6
AUC : 0.872
AIC : 61.5
n : 60

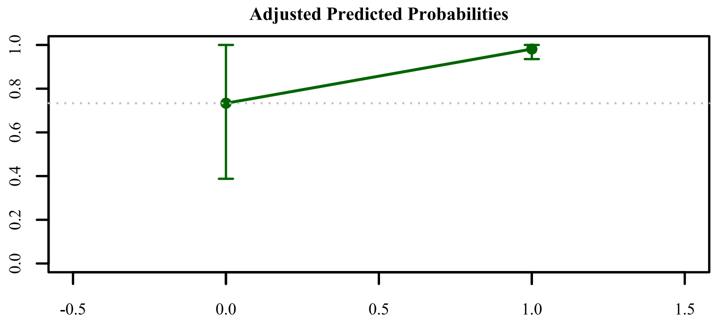

Leukocytes (Scaled)

Univariate Model

β : 0.83
95% CI : [-0.21, 1.87]
OR* : 2.29 [0.81–6.50]
p-value : 0.1185

AUC : 0.617
n : 60

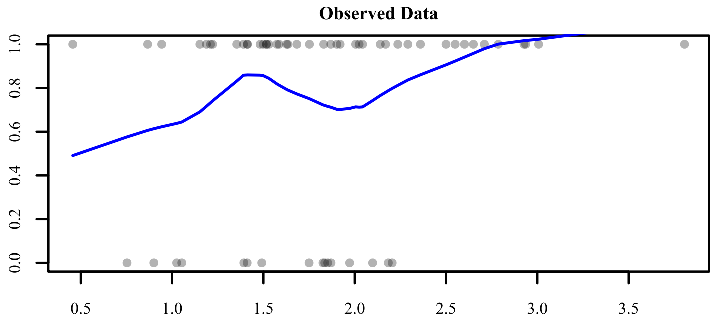

Multivariate Model

Adj β : 0.52
95% CI : [-1.01, 2.06]
Adj OR* : 1.69 [0.36–7.84]
p-value : 0.5042
Covariates: 6
AUC : 0.791
AIC : 69.7
n : 60

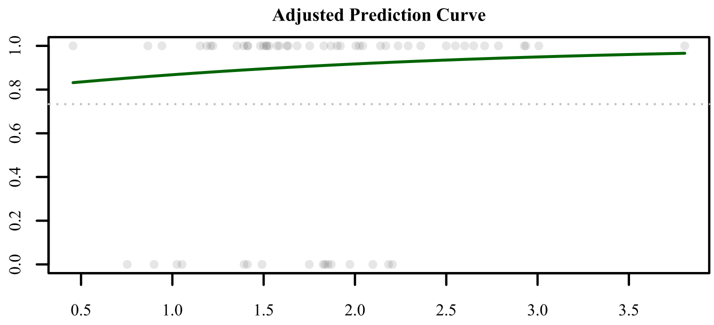

Lactate

Univariate Model

β : -0.03

95% CI : [-0.26, 0.20]

OR* : 0.97 [0.77–1.22]

p-value : 0.8109

AUC : 0.540

n : 60

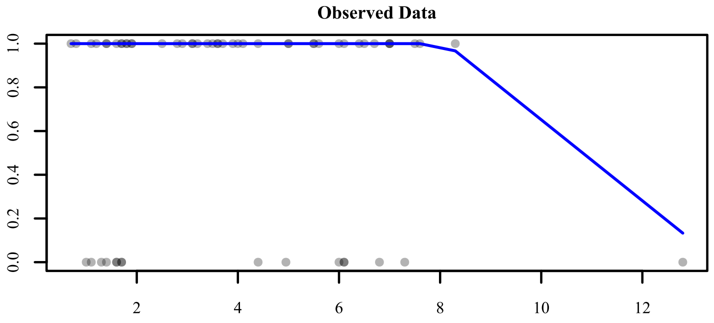

Multivariate Model

Adj β : -0.18

95% CI : [-0.52, 0.16]

Adj OR* : 0.84 [0.59–1.18]

p-value : 0.3085

Covariates: 6

AUC : 0.791

AIC : 69.7

n : 60

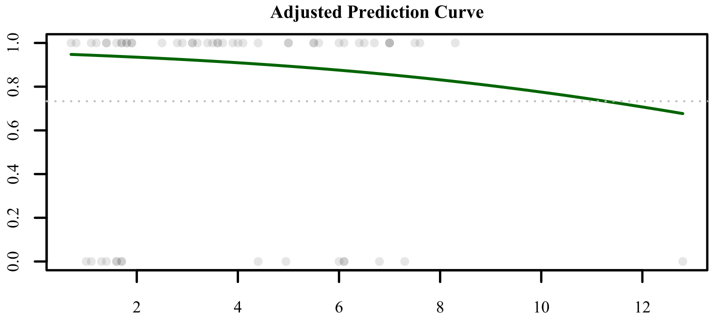

pH (Scaled)

Univariate Model

β : 0.56

95% CI : [-0.17, 1.28]

OR* : 1.74 [0.85–3.59]

p-value : 0.1326

AUC : 0.632

n : 58

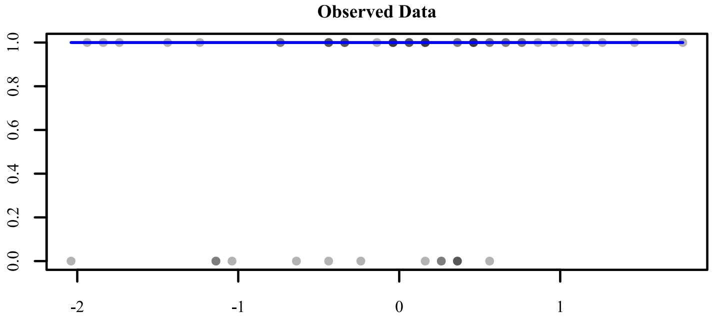

Multivariate Model

Adj β : 1.28

95% CI : [-0.00, 2.56]

Adj OR* : 3.59 [1.00–12.89]

p-value : 0.0502

Covariates: 6

AUC : 0.831

AIC : 59.6

n : 58

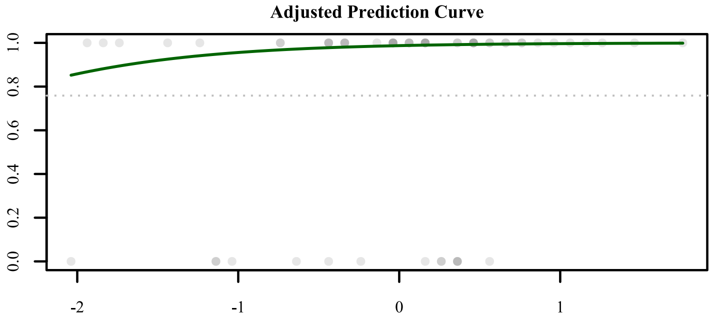

Glycemia

Univariate Model

β : 0.01
95% CI : [-0.00, 0.01]
OR* : 1.01 [1.00–1.02]
p-value : 0.1202

AUC : 0.610
n : 60

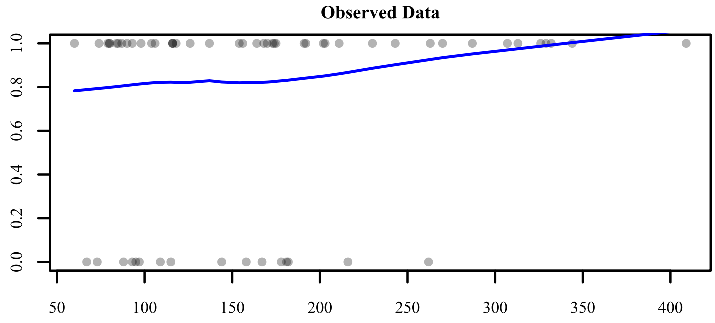

Multivariate Model

Adj β : 0.01
95% CI : [-0.00, 0.03]
Adj OR* : 1.01 [1.00–1.03]
p-value : 0.1155
Covariates: 6
AUC : 0.791
AIC : 69.7
n : 60

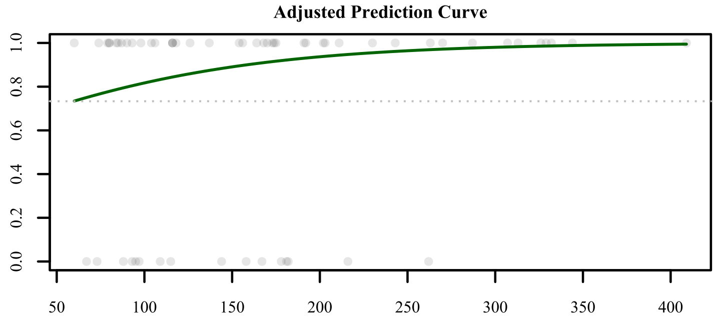

Platelets (Scaled)

Univariate Model

β : 0.26
95% CI : [-0.28, 0.81]
OR* : 1.30 [0.76–2.24]
p-value : 0.3382

AUC : 0.582
n : 60

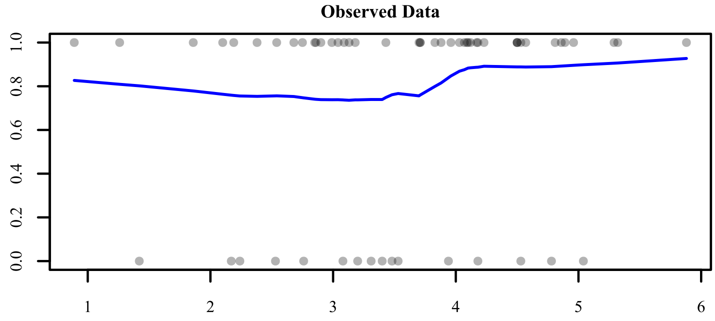

Multivariate Model

Adj β : -0.04
95% CI : [-0.76, 0.68]
Adj OR* : 0.96 [0.47–1.97]
p-value : 0.9079
Covariates: 6
AUC : 0.791
AIC : 69.7
n : 60

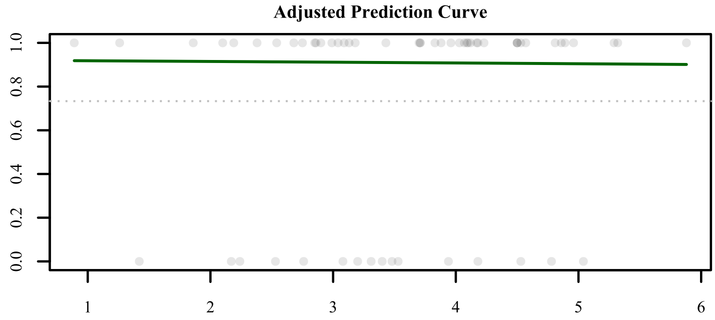

Sodium

Univariate Model

β : 0.04

95% CI : [-0.13, 0.22]

OR* : 1.05 [0.88–1.25]

p-value : 0.6212

AUC : 0.548

n : 60

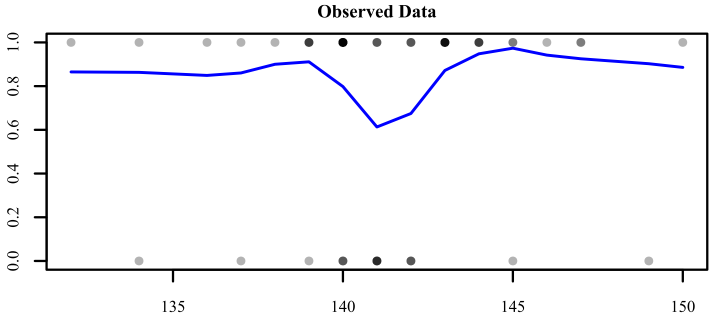

Multivariate Model

Adj β : -0.05
95% CI : [-0.27, 0.16]
Adj OR* : 0.95 [0.76–1.18]
p-value : 0.6229
Covariates: 6
AUC : 0.793
AIC : 71.4
n : 60

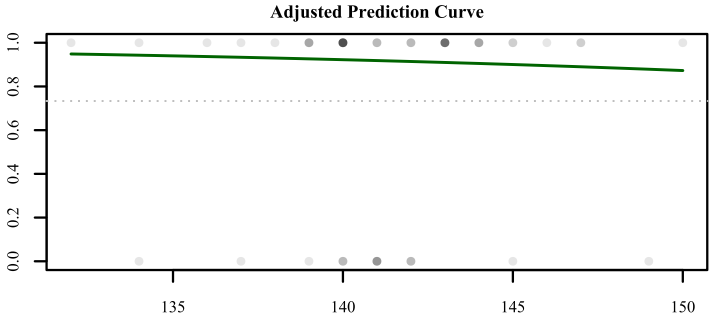

Potassium

Univariate Model

β : -0.30

95% CI : [-1.03, 0.42]

OR* : 0.74 [0.36–1.53]

p-value : 0.4113

AUC : 0.592

n : 60

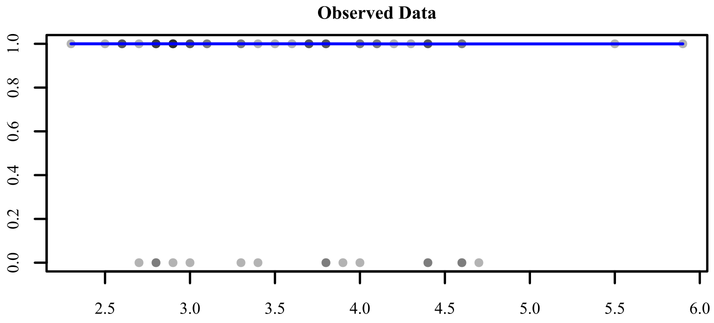

Multivariate Model

Adj β : -0.64
95% CI : [-1.86, 0.57]
Adj OR* : 0.53 [0.16–1.77]
p-value : 0.2980
Covariates: 6
AUC : 0.808
AIC : 70.5
n : 60

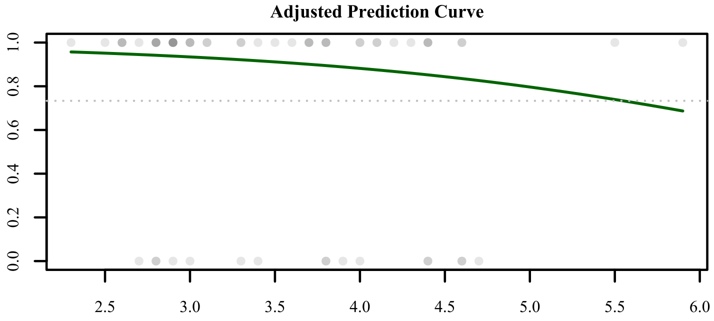

Troponin (scaled)

Univariate Model

β : 0.77

95% CI : [-0.15, 1.68]

OR* : 2.15 [0.86–5.36]

p-value : 0.0997

AUC : 0.731

n : 60

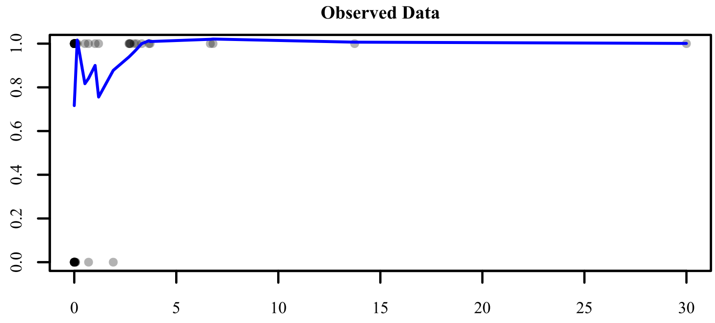

Multivariate Model

Adj β : 0.78

95% CI : [-0.19, 1.75]

Adj OR* : 2.19 [0.83–5.76]

p-value : 0.1131

Covariates: 6

AUC : 0.791

AIC : 69.7

n : 60

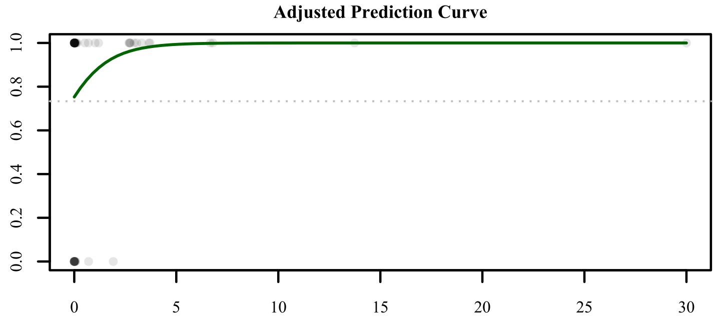

Total CK (scaled)

Univariate Model

β : 14.99

95% CI : [-7.34, 37.31]

OR* : 3223683.47 [0.00–159833353

p-value : 0.1883

AUC : 0.615

n : 60

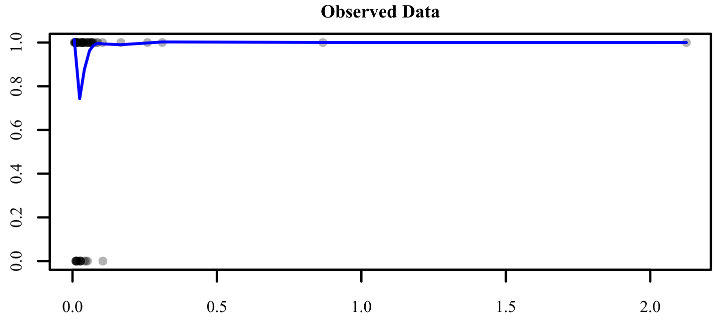

Multivariate Model

Adj β : 5.53

95% CI : [-16.39, 27.44]

Adj OR* : 251.06 [0.00–823228257552

p-value : 0.6211

Covariates: 6

AUC : 0.791

AIC : 69.7

n : 60

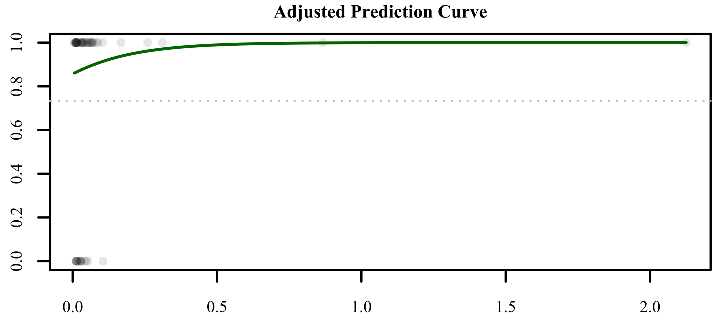

Amylase

Univariate Model

β : 0.01
95% CI : [-0.00, 0.01]
OR* : 1.01 [1.00–1.01]
p-value : 0.1161

AUC : 0.625
n : 45

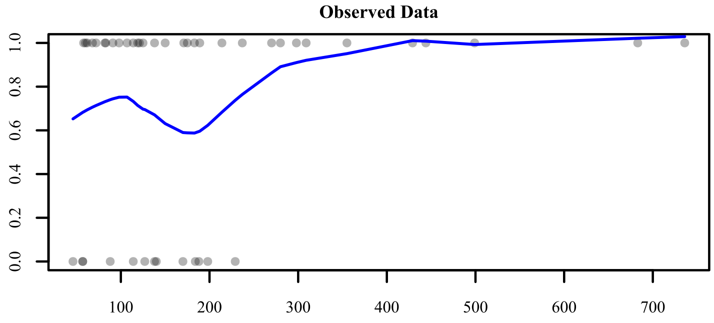

Multivariate Model

Adj β : 0.00
95% CI : [-0.01, 0.01]
Adj OR* : 1.00 [0.99–1.01]
p-value : 0.6603
Covariates: 6
AUC : 0.877
AIC : 48.9
n : 45

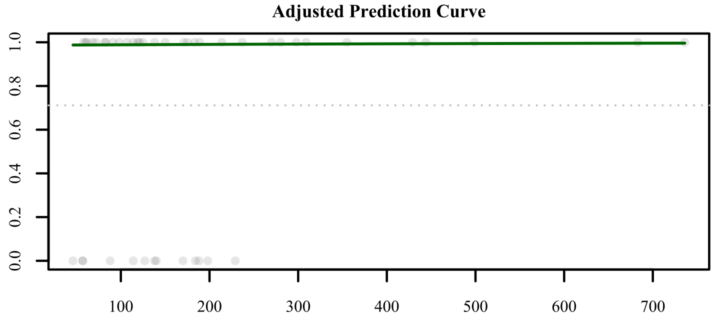

Bicarbonate

Univariate Model

β : -0.19
95% CI : [-0.39, 0.01]
OR* : 0.83 [0.68–1.01]
p-value : 0.0689

AUC : 0.666
n : 58

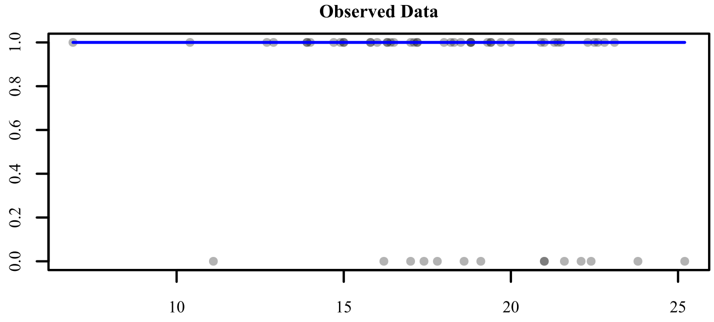

Multivariate Model

Adj β : -0.25
95% CI : [-0.59, 0.09]
Adj OR* : 0.78 [0.56–1.09]
p-value : 0.1513
Covariates: 6
AUC : 0.817
AIC : 62.6
n : 58

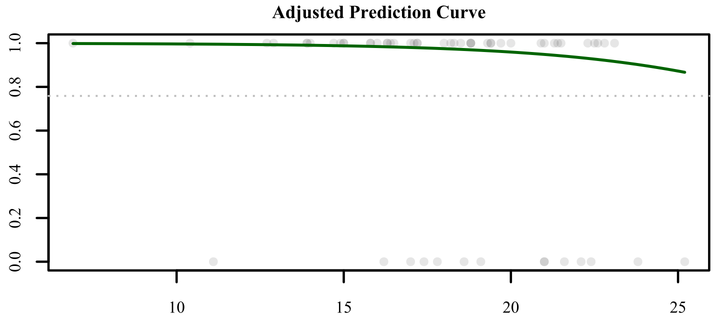

Cardiac Dysfunction

Univariate Model

OR : 1.53
95% CI : [0.27, 8.63]
p-value : 0.6277

P(Intensive Care|0): 0.833
P(Intensive Care|1): 0.885

AUC : 0.553
n : 44

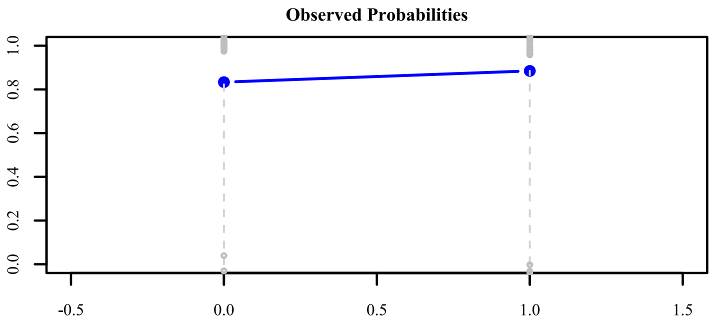

Multivariate Model

Adj OR : 0.71
95% CI : [0.07, 7.73]
p-value : 0.7782
Covariates: 6
AUC : 0.772
AIC : 45.4
n : 44

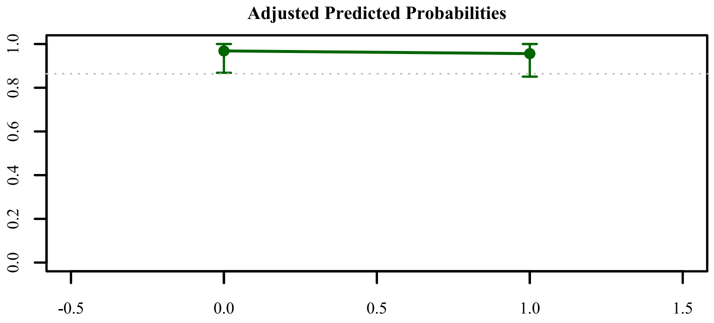

1.4 Supplementary Figure 4S: Residual Analyses – QQ Plots, Shapiro-Wilk and Kolmogorov-Smirnov Tests

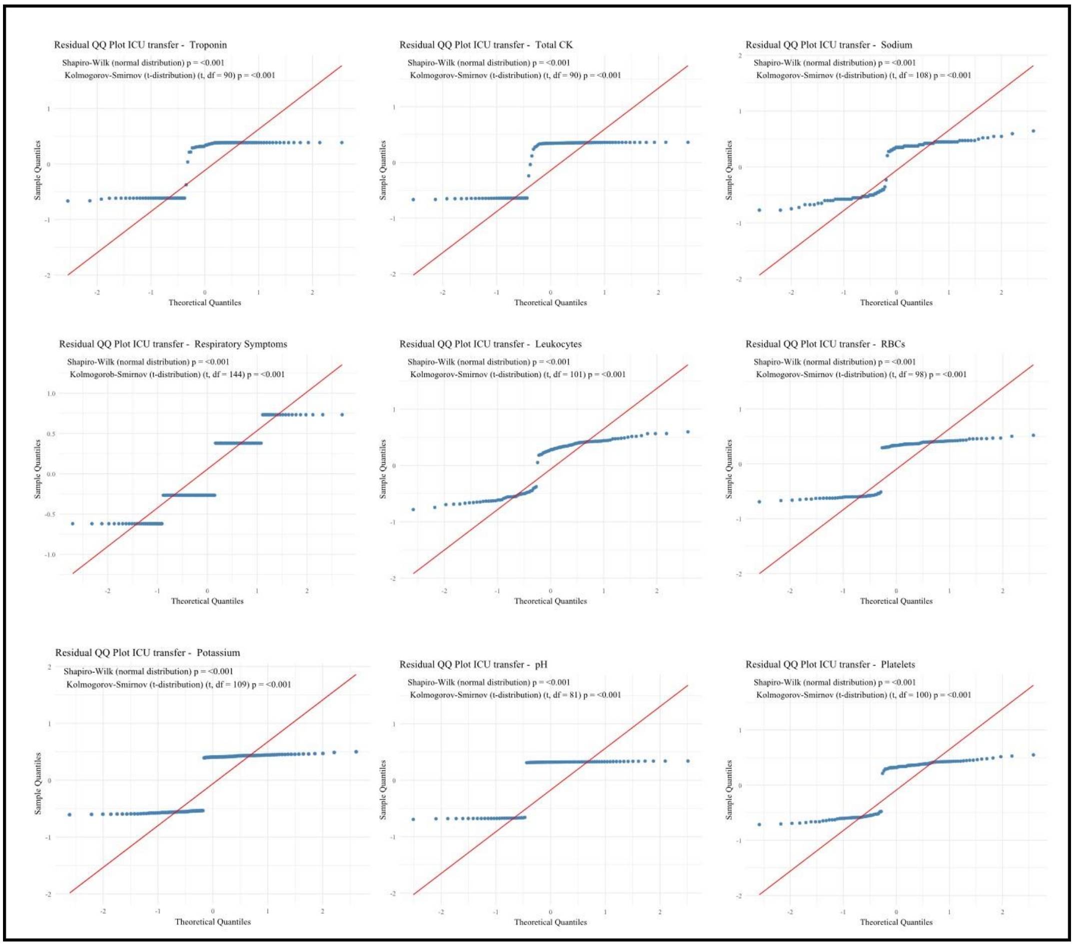

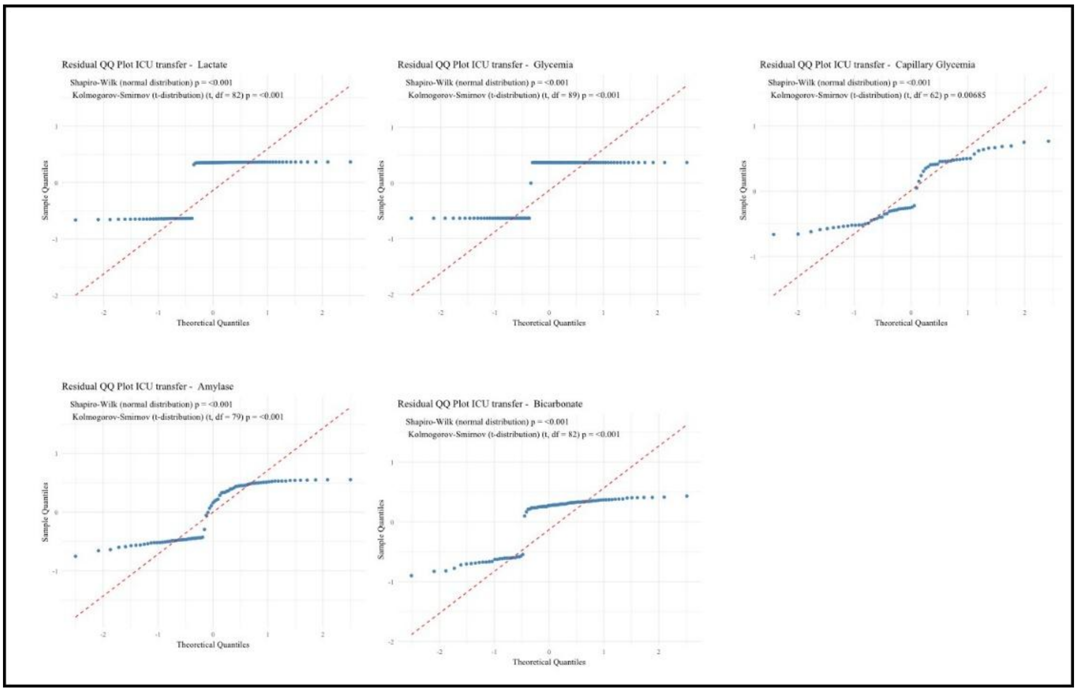

*ICU: Intensive Care Unit; df: degrees-of-freedom for t-distribution.

1.5 Supplementary Figures 5S: Univariate and Multivariate Regressions – Death or Sequelae

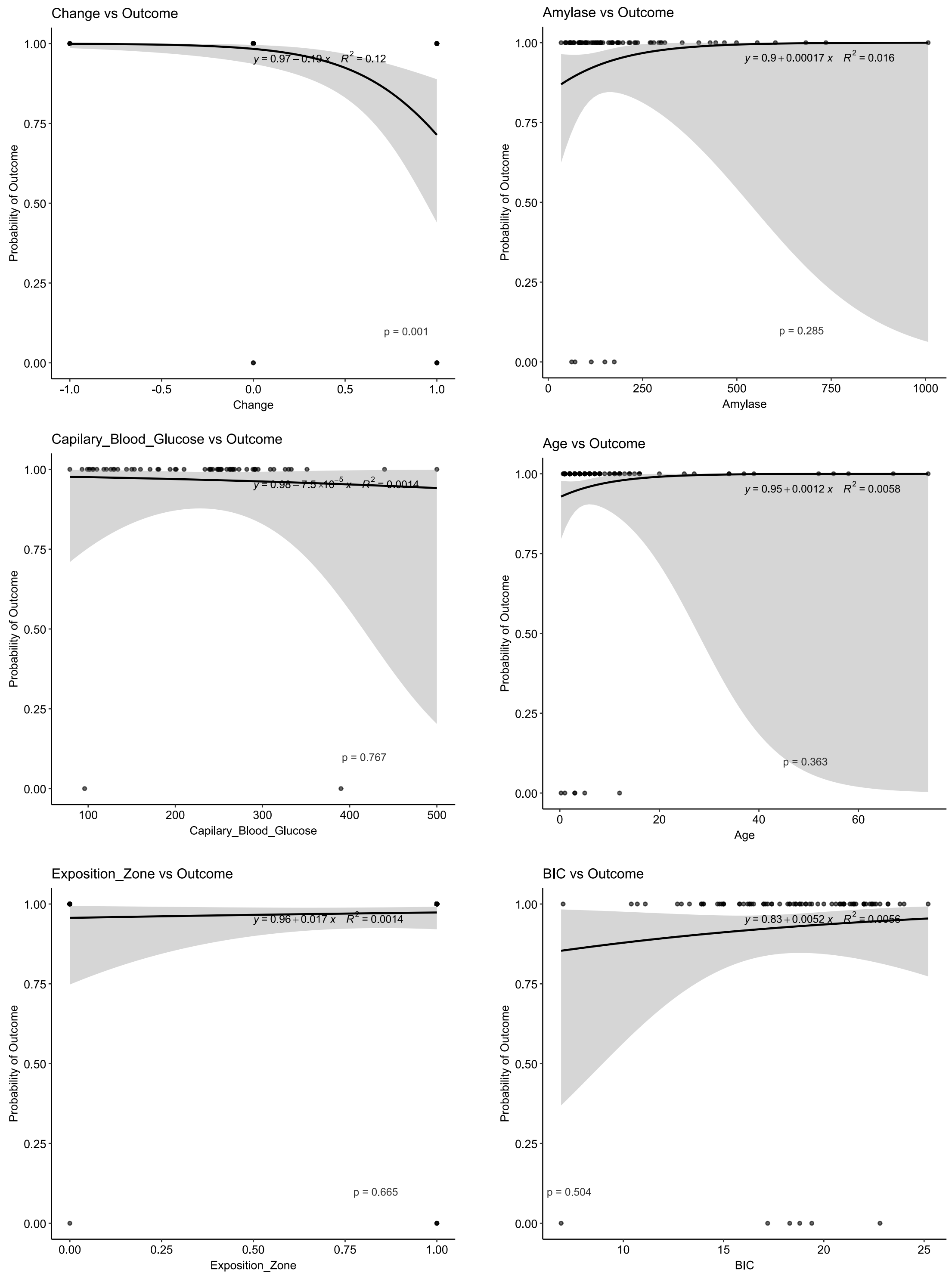

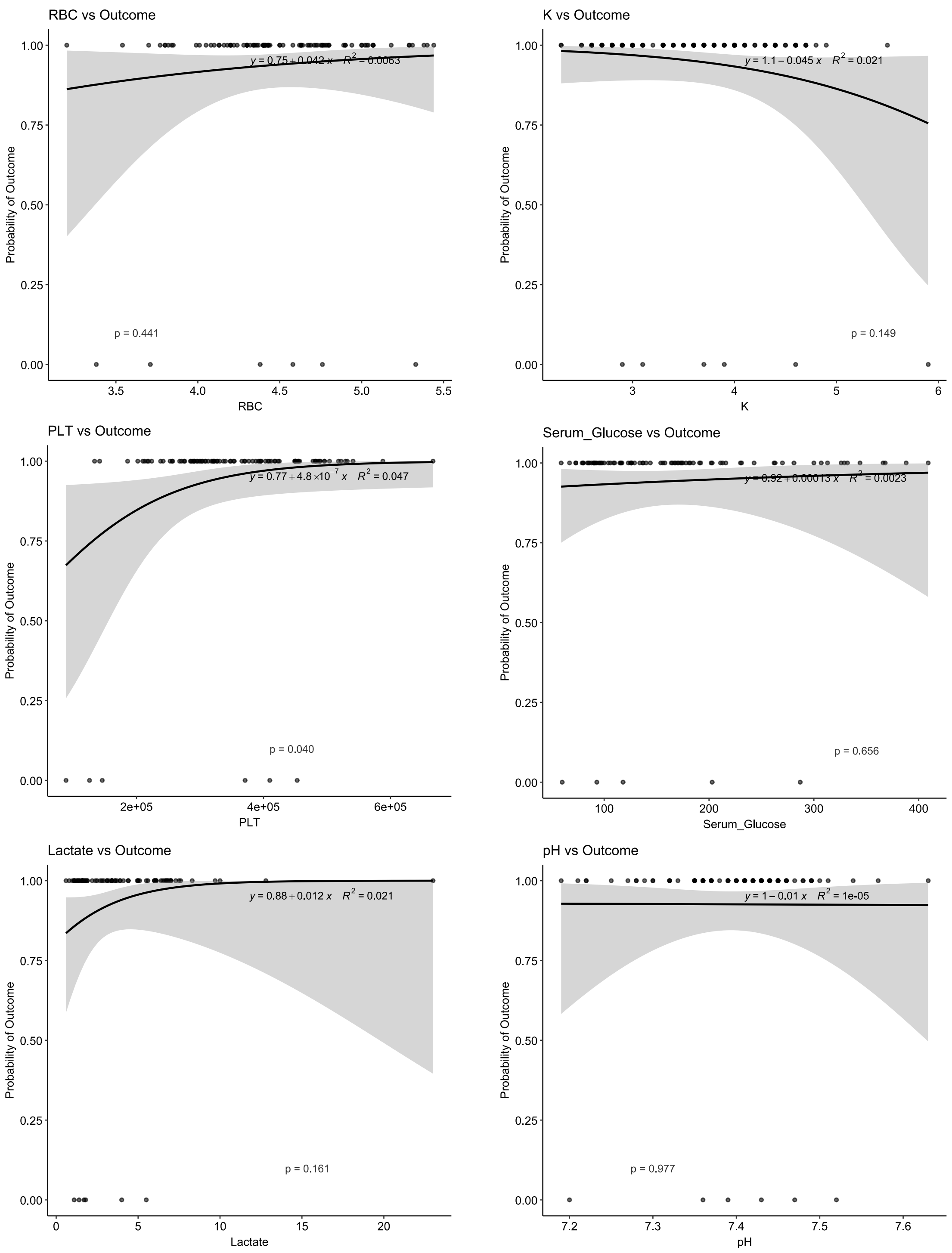
RBC vs Outcome
y = 0.75 + 0.042 x   R2 = 0.0063
p = 0.441
Probability of Outcome
RBC
K vs Outcome
y = 1.1 − 0.045 x   R2 = 0.021
p = 0.149
Probability of Outcome
K
PLT vs Outcome
y = 0.77 + 4.8 ×10−7 x   R2 = 0.047
p = 0.040
Probability of Outcome
PLT
Serum_Glucose vs Outcome
y = 0.92 + 0.00013 x   R2 = 0.0023
p = 0.656
Probability of Outcome
Serum_Glucose
Lactate vs Outcome
y = 0.88 + 0.012 x   R2 = 0.021
p = 0.161
Probability of Outcome
Lactate
pH vs Outcome
y = 1 − 0.01 x   R2 = 1e-05
p = 0.977
Probability of Outcome
pH

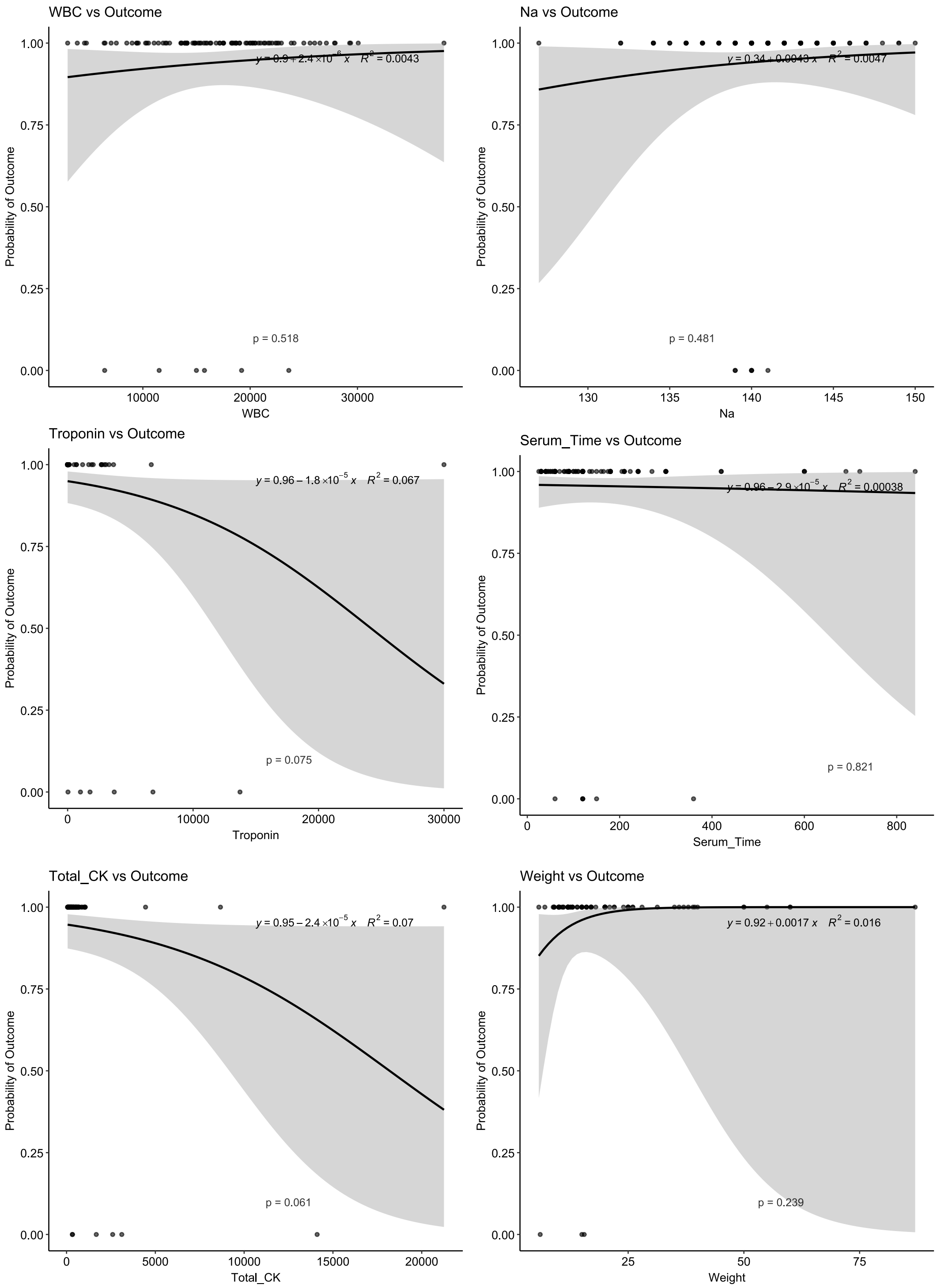
WBC vs Outcome
Probability of Outcome
WBC
p = 0.518
R^2 = 0.0043
Na vs Outcome
Na
p = 0.481
Troponin vs Outcome
y = 0.96 – 1.8 ×10^−5 x R^2 = 0.067
Troponin
p = 0.075
Serum_Time vs Outcome
R^2 = 0.00038
Serum_Time
p = 0.821
Total_CK vs Outcome
y = 0.95 – 2.4 ×10^−5 x R^2 = 0.07
Total_CK
p = 0.061
Weight vs Outcome
y = 0.92 + 0.0017 x R^2 = 0.016
Weight
p = 0.239

2. Supplementary Tables

2.1 Supplementary Table 1S: Variable List

**Variable**
**Symptoms** (one variable each): pain, tremors, sweating, paleness, nausea, vomiting, sialorrhea, abdominal pain, irritability, drowsiness, cold extremities, tachypnea, bradycardia, tachycardia, dyspnea, rales, low oxygen saturation, hypotension, hypertension, circulatory shock, altered mental status, convulsion, fever, arrhythmias, cardiopulmonary arrest, acute lung edema, infection.
**Laboratory findings** (one variable each): capillary blood glucose, venous blood glucose, leukocytes, erythrocytes, platelets, sodium, potassium, bicarbonate, amylase, pH, lactate, troponin, total CK,
**Imaging:** Cardiac dysfunction on echocardiogram.
**Primary outcome:** Intensive care unit transfer
**Secondary outcomes** (one variable each): intubation, death, sequelae.

A total of 46 variables were included in this analysis, after duplicate variables were discarded.

2.2 Supplementary Table 2S: Signs and Symptoms at Initial Presentation – Survival Analysis

Variables (%)	All patients (n = 137)*	Survivors (n = 129)	Nonsurvivors o with Severe Disability (n = 6	p-value**
**Vomiting**	90	87	2	0.1799
**Nausea**	19	18	1	1
**Abdominal Pai**	15	15	0	1
**Cold Extremitie**	5	5	0	1
**Respiratory Insufficiency**	5	5	0	1
**Tachypnea**	32	29	2	0.6205
**Irritability**	2	2	0	1
**Lethargy/ Drowsiness**	22	22	0	0.5889
**Salivation**	19	19	0	0.5943
**Wheezing**	1	1	0	1
**Diffuse Sweatin**	52	49	2	1
**Sphincter Relaxation**	1	1	0	1
**Myosis**	1	1	0	1
**Muscle Spasms**	1	1	0	1
**Oliguria**	1	1	0	1
**Altered Level o Consciousness**	6	6	0	1
**Hypotension**	6	5	0	1
**Hypertension**	7	7	0	1
**Acute Pulmonar Edema**	15	12	3	**0.0183**
**Bradycardia**	25	24	1	1
**Tachycardia**	27	26	1	1
**Palpitations**	1	1	0	1

**Hypothermia**	3	3	0	1
**Hyperthermia**	1	1	0	1
**Cardiorespirator Arrest**	1	1	0	1
**Rash**	1	1	0	1
**Hypotonia**	1	1	0	1
**Dizziness**	1	1	0	1
**Paresthesia**	1	1	0	1

*Clinical presentation data not available for 9 patients, with 6 in the Survivors group.
**Hypothesis tests comparing the groups were conducted by Fisher's exact tests.

2.3 Supplementary Table 3S: Table 1 with mean and standard deviation data

Table 1. Baseline Characteristics

Variable	All patients	ICU Transfer+		p-values	Complete recovery*		p-values
	(n = 146)	Yes (n = 64)	No (n = 80)		Yes (n = 135)	No (n = 6)	
Age (mean [SD])	8,448 [12,191]	4,763 [3,867]	11,407 [15,521]	0,006	8,675 [12,575]	4,041 [4,243]	0,1998
Sex (n)							
Male	71	26	46	0,064	69	2	0,6807
Female	70	38	34		66	4	
Weight (mean [SD]) (n = 79)**	21,869 [14,752]	20,771 [16,309]	23,0 [13,107]	0,30	22,446 [14,982]	10,5 [6,363]	0,2252
Initial Severity (n)							
Low	5	0	5	< 0.001	5	0	0.3440

**Intermediate**	40	9	30		39	0	
**High**	101	55	45		91	6	
**Exposition Area (n)**							
**Urban**	118	51	65	0,499	110	3	0,5277
**Rural**	23	12	11		22	1	
**Time to Anti-scorpionic Serum (n = 141)+ (mean [SD])**	141,560 [136,84]	136,714 [122,396]	145,355 [149,792]	0,891	141,923 [140,373]	155,0 [104,642]	0,2858

*5 patients had no information regarding sequelae-free survival. ** Weight was only available for 79 patients. + Two patients had no information regarding ICU transfer. Two patients had no information regarding time to serum. **In this analysis, data distribution was not considered and only Student's t tests were conducted.** Fisher's exact tests were conducted for discrete data.

2.4 Supplementary Table 4S: Table 2 with mean and standard deviation data

Variable	All patients	ICU Transfer^{+}		p-values	Complete recovery*		p-values
	(n = 146)	Yes (n = 64)	No (n = 80)		Yes (n = 135)	No (n = 6)	
RBCs (n = 101)	4,484 [0,457]	4,55 [0,461]	4,409 [0,447]	0,284	4,505 [0,442]	4,356 [0,711]	0.6683
WBCs (n = 103)	16765,728 [6457,355]	18043 [6391]	14876 [6170]	0,025	16.999,891 [6501,670]	15.248,333 [5952,506]	0.5988
PLT (n = 102)	357313 [108367]	378847 [103774]	329452 [109484]	0,043	364.945,055 [104.359,567]	265.833,333 [162.516,973]	0.1753
K^{+} (n = 111)	3,613 [0,729]	3,576 [0,717]	3,654 [0,754]	0,554	3,563 [0,705]	4,016 [1,103]	0.3210
Na^{+} (n = 110)	140,836 [3,683]	140,655 [2,875]	140,941 [4,425]	0,629	140,939 [3,848]	139,833 [0,752]	0.1919
BIC	18,308 [3,777]	17,893 [3,565]	18,762 [4,054]	0,155	18,312 [3,686]	17,233 [5,404]	0.8710

**(n = 84)**							
**Total CPK (n = 93)**	915,271 [2778,454]	413,774 [485,81]	1521,763 [4192,795]	0,646	736,865 [2523,299]	3690,833 [5229,741]	0.004
**Troponin (n = 102)**	964,796 [3447,676]	729,914 [2127,829]	1281,613 [4688,016]	0,852	750,573 [3303,713]	4515,233 [5113,654]	0.0012
**Amylase (n = 87)**	192,839 [174,876]	221,652 [196,802]	154,971 [134,521]	0,090	203,930 [182,088]	114,4 [48,911]	0.3210
**Lactate (n = 84)**	4,192 [3,281]	4,138 [2,316]	3,751 [2,845]	0,209	4,41 [3,370]	2,583 [1,761]	0.1367
**pH (n = 84)**	7,395 [0,082]	7,41 [0,072]	7,376 [0,091]	0,074	7,394 [0,081]	7,395 [0,111]	0.7520
**Serum Glucose (n = 97)**	165,703 [86,059]	163,462 [80,829]	168,692 [93,576]	0,913	169,975 [86,724]	152,2 [92,096]	0.6419
**Capillary Glucose (n = 64)**	221,765 [90,445]	230,75 [90,251]	212,781 [91,175]	0,354	223,915 [87,674]	243 [207,889]	1

*5 patients had no information regarding sequelae-free survival. **In this analysis, data distribution was not considered and Student’s t tests were used for all continuous variables.**

2.5 Supplementary Table 5S: Statistical Power Table:

Statistical power (1- β)	Spearman's ρ	Sample size (n)*
80%	0.3	85
80%	0.25	124
80%	0.2	194
90%	0.3	113
90%	0.25	165
95%	0.3	139
95%	0.25	203

*Formula:

$$n = \frac{(Z_{1-\alpha/2} + Z_{1-\beta})^2}{\left(0.5 \cdot \ln \frac{1+r}{1-r}\right)^2} + 3$$

n: sample size;

r: expected correlation coefficient under alternative hypothesis;

$Z_{1-\alpha/2}$: Critical z-value for two-tailed test at level α (generally 1.96);

$Z_{1-\alpha/2}$: Critical z-value for chosen statistical power;

3. Model Variables:

Model 1: covariates: cardiac dysfunction on echocardiogram or POCUS, respiratory symptoms, cardiac symptoms, neurologic symptoms, gastrointestinal symptoms, pH, white blood cells, platelets, glycemia; AIC: 59.0; AUC: 0.84.

Model 2: covariates: potassium, red blood cells, platelets, amylase, bicarbonate, troponin, total CPK; AIC: 55.5; AUC: 0.80.

Model 3: covariates: time to serum, platelets, red blood cells, troponin; AIC: 109.4; AUC: 0.69.

Model 4: covariates: cardiac dysfunction on echocardiogram or POCUS, cardiac symptoms, neurologic symptoms, gastrointestinal symptoms, white blood cells, platelets, troponin; AIC: 43.6; AUC: 0.70.

Models 5: covariates: cardiac dysfunction on echocardiogram or POCUS, cardiac symptoms, neurologic symptoms, gastrointestinal symptoms, glycemia, troponin, total CPK, platelets; AIC 42.3; unadjusted AUC: 0.90; adjusted AUC: 0.65.

Model 6: same covariates as model 5; AIC: [159.31-177.4]; AUC: [0.77-0.84]. (Range across five imputations).

Model 7: covariates: cardiac dysfunction on echocardiogram or POCUS, tachypnea, cold extremities, irritability, drowsiness, pulmonary crepitus, vomiting, abdominal pain, sweating, tremors, tachycardia, bradycardia, hypertension, hypotension, low oxygen saturation, sodium, amylase, glycemia, troponin, lactate, white blood cells; AIC: 137; AUC: 0.93.

Simplified model 7: covariates: cardiac dysfunction on echocardiogram or POCUS, tachypnea, cold extremities, drowsiness, pulmonary crepitus, vomiting, abdominal pain,

sweating, tachycardia, bradycardia, hypotension, low oxygen saturation, sodium, amylase, lactate; AIC: 130.2; AUC: 0.92. This model had the optimal cut-off point of 0.567 (calculated probability), corresponding to a specificity of 0.95 and a sensitivity of 0.78.

4. Nomogram experimental multimedia example

The interactive nomogram example, created with AI assistance, can be accessed at:

https://gemini.google.com/share/63cb77f4d1f1

This is also an example of how a clinical score can be turned into a more practical and execution-ready application, if its clinical utility is of interest.
